# The Effect of Branched Alkyl Chain Length on the Properties of Supramolecular Organogels from Mono-*N*-Alkylated Primary Oxalamides

**DOI:** 10.3390/gels9010005

**Published:** 2022-12-22

**Authors:** Khalid Azyat, Darren Makeiff, Bradley Smith, Mickie Wiebe, Steve Launspach, Ashley Wagner, Marianna Kulka, Nicolas Godbert

**Affiliations:** 1 Nanotechnology Research Center, National Research Council of Canada, 11421 Saskatchewan Drive, Edmonton, AB T6G 2M9, Canada; 2 Dipartimento di Chimica e Tecnologie Chimiche, Università della Calabria, 87036 Arcavacata di Rende, CS, Italy

**Keywords:** supramolecular gel, organogel, low molecular weight gelator, self-assembly, oxalamide, H-bonding

## Abstract

Mono-*N*-alkylated primary oxalamide derivatives with different sized branched alkyl tail-groups were excellent low molecular weight gelators for a variety of different organic solvents with different polarities and hydrogen-bonding abilities. Solvent-gelator interactions were analyzed using Hansen solubility parameters, while ^1^H NMR and FTIR spectroscopy were used to probe the driving forces for the supramolecular gelation. The molecular structures of the twin tail-groups did not significantly affect the supramolecular gelation behavior in different solvents. However, for select solvents, the molecular structures of the tail-groups did have a significant effect on gel properties such as the critical gelator concentration, thermal stability, gel stiffness, gel strength, network morphology, and molecular packing. Finally, metabolic activity studies showed that the primary alkyl oxalamide gelators had no effect on the metabolic activity of mouse immune cells, which suggests that the compounds are not cytotoxic and are suitable for use in biomedical applications.

## 1. Introduction

Supramolecular gels from low molecular weight gelators (LMWGs) [1,2,3] are a class of soft functional materials with versatile properties that have recently garnered significant scientific interest for numerous applications including water purification [4,5], drug delivery [6], sensing [7,8], 3D printing [9], tissue engineering [10], as well as many others [11,12]. The hierarchical self-assembly of LMWG molecules results in anisotropic structures, most commonly fibers, which form physical networks capable of immobilizing the solvent by surface tension and capillary forces [13]. Immobilization of solvent molecules within the network pores culminates in a fascinating transformation from a solution to a viscoelastic solid, in many cases at remarkably low gelator concentrations [14]. Furthermore, in contrast to permanently covalently cross-linked polymer gels, supramolecular gels are physical gels, which can be formed reversibly in the presence of various types of stimuli such as heat, light, chemicals, ultrasound, and mechanical sheer [15,16,17]. The formation of the 3D network is driven by non-covalent interactions such as hydrogen-bonding (H-bonding), π-π stacking, van der Waals and metal-ligand interactions [1,2,3,18].

Despite the broad range of chemical structures of organic molecules known form supramolecular gels to date, the rational design of LMWGs for gelating specific liquids to give supramolecular gels with suitable properties for a certain application [3,13,19,20] remains a significant challenge. Towards this goal, many studies have been carried out in order to better understand the influence of molecular structure on the gelator self-assembly process and gel properties, which involve the effect of slight structural changes on the gelation and gel properties for LMWGs such as hydroxystearic acids [21], glucosamines [22], stearates [23], stearamides [24], cholesterols [25], bile acids [26], coumarins [27], ureas [28], carbamates [29], alkylated amino acids [30], polyaromatics [31], dendritic supramolecules [32], as well as peptide-based hydrogelators [33] to name a few. Such studies have revealed that gel properties such as gelation ability with different solvents, critical gel concentration (CGC), gel strength and thermal stability can be modulated with minimal structural changes, without modifying the types of intermolecular interactions that drive the gel formation in a specific solvent allowing fine-tuning between gel properties and related applications [24].

**Scheme 1:**
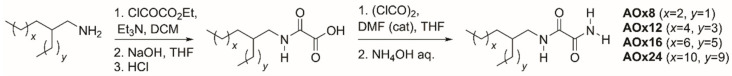
Synthesis of branched alkylated primary oxalamide gelators.

Oxalamide compounds are of considerable interest due to their potential in biomedical applications [34,35,36,37,38,39,40,41,42,43,44,45], catalysis [46,47,48], foods [49], and self-assembling behavior to form gels [50,51,52,53,54,55,56]. The oxalamide functional group consists of two amide groups next to each other in reverse mode, connected by two consecutive carbonyl groups, which has been extensively used as supramolecular synthon to generate LMWGs [50,51,52,53,54,55,56,57,58]. A variety of bis, homo and hetero-functionalized bolaform, oxalamide-based gelators have been reported over the past two decades [50,51,52,53,54,55,56], while only two reports of mono-functionalized, primary oxalamide gelators exist to date [57,58]. Thus the present study aims to report new LMWGs based on mono-*N*-alkylated primary oxalamides (Scheme 1) and evaluate the effect of different sized, branched tail-groups on the supramolecular gelation behavior and gel properties. These molecules possess twin alkyl tail-groups with A and B hydrocarbon chains ranging from 6–14 and 2–10 C’s, respectively, attached to the H-bonding oxalamide head-group. Herein, we discuss the solubility/gelation behavior in 31 different solvents via the tube inversion test and analysis of the results using Hansen solubility parameters (HSPs). ^1^H NMR and FTIR spectroscopies were used to probe the driving forces for the self-assembly of these LWMGs. Additionally, the effect of tail-group structure on various gel properties (i.e., critical gel concentration (CGC), thermal stability, gel network stiffness, mechanical strength, morphology, and molecular packing) is discussed. Finally, the cytotoxicity of these compounds was also assessed in order to evaluate their potential use for biomedical applications.

## 2. Results and Discussion

### 2.1. Synthesis

All four primary oxalamide compounds were synthesized in three steps from the corresponding alkylamines in good yield without requiring tedious chromatography purification steps. The alkylamine precursor for AOx8 was obtained from a commercial source, while the amines for AOx12, AOx16 and AOx24 were obtained via reduction of the corresponding amide derivatives using lithium aluminum hydride. First, the alkyl amines were converted to the corresponding oxalic acid ethyl esters via reaction with chloroethyl oxoacetate [59], followed by saponification using NaOH in THF to yield the corresponding oxalic acids (Scheme 1). The oxalic acids were then converted to the corresponding acid chlorides, which were then reacted with ammonium hydroxide to afford the desired alkylated primary oxalamides in good yields (Scheme 1). The chemical structures of all four oxalamides were confirmed using ^1^H and ^13^C NMR spectroscopy and high resolution mass spectrometry (HRMS) (Figures S1–S4, ESI).

### 2.2. Gelation Behavior

Recently, we have reported that branched (Guerbet-type) hydrocarbons are effective tail-groups for LMWGs based on isophthalic acids [60] and benzimidazolones [61]. The organogelation capacity for all four oxalamides in Scheme 1 was probed in 31 organic liquids (Table S1). AOx8, AxO12, AOx16, and AOx24 were all found to be exceptional gelators, gelling 23, 23, 20, and 24 organic liquids, respectively. All four of the gelators gelled similar solvents, which included low polarity solvents (i.e., hexanes, toluene, etc…), polar solvents (i.e., acetonitrile, acetone, ethyl acetate), protic polar solvents (i.e., methanol, benzyl alcohol, propylene glycol (PG), and ethanol amine), as well as fatty acids/organic acids/long chain esters (i.e., isopropyl myristate, olive oil, corn oil, soybean oil, sesame oil, castor oil). Overall, tail-group structure had very little effect on the gelation behavior, with a few exceptions. For example, AOx16 was the only compound that did not form gels with benzyl alcohol, acetone and ethyl acetate, while AOx8 was the only compound that did gel chloroform, but did not gel DMF or lactic acid. Interestingly, AOx24 was the only compound to form a gel at a higher gelator concentration (i.e., 3.5 wt %) with DMSO, which is well-known to be highly effective at disrupting H-bonded aggregates. All four compounds were also “supergelators” for 10, 14, 16 and 19 solvents, respectively, forming gels at gelator concentrations below 1 wt % [62,63]. 

Despite gelling similar solvents, the CGC was dependent on twin tail-group structure. For most of the solvents, the CGC generally decreased with increasing twin tail-group size. For example, the CGCs for gels with hexadecane (HD) were 0.7, 0.05, <0.05 and <0.05 wt % for AOx8, AOx12, AOx16 and AOx24, respectively (35, 2, <2 and <1 in mM, respectively, Table S1). Only the solvents cinnamaldehyde, ethanolamine and methanol had one gelator that deviated from this trend. Overall, AOx24 was regarded as the most effective gelator, exhibiting slightly better versatility in forming gels with the most solvents, and efficiency, in exhibiting the lowest CGCs and behaving as a supergelator with the most solvents.

### 2.3. Hansen Solubility Parameters

**Figure 1:**
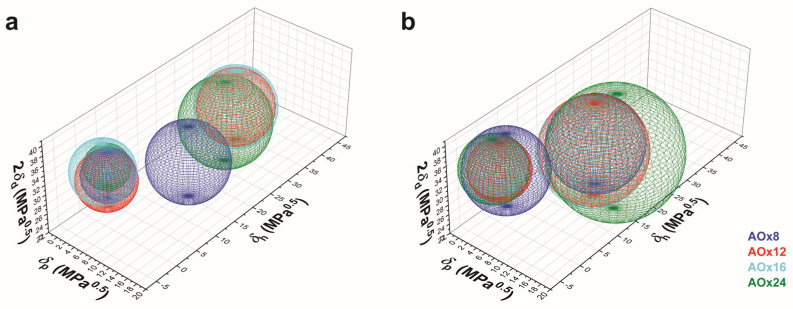
Gelation spheres for alkyloxalamide gelators at (**a**) 1 wt % and (**b**) 3.5 wt %.

Solvent effects are known to play a significant role in the ability of small molecules to undergo hierarchical self-assembly into 3D network structures. Gel formation in a particular solvent is a consequence of a delicate equilibrium between the solubilization and crystallization of the small gelator molecules. Hansen solubility parameters (HSPs) have been demonstrated to be valuable computational tools to provide insight into complex solvation effects or predicting the gelation behavior of LWMGs [64,65,66]. For this study, HSPs were used to gain further insight into the effect of solvents with different polarities (δ_p_) and abilities to form dispersive (δ_d_) and H-bonding interactions (δ_h_) with the four LMWGs reported here. Figure 1 shows the gelation spheres for all four gelators in 3D Hansen space. The best fit for all gel tests at 1 and 3.5 wt % in 28 solvents (Table S2) was obtained using two gel spheres at low and high δ_p_/δ_h_ values (Figure 1 and Figure S5). The existence of two gelation spheres has been previously reasoned to be associated with two different types of crystalline packing [67]. 

At 1 wt %, the HSPs of the gel spheres at low δ_p_/δ_h_ values did not change significantly, while the HSPs for the gel spheres at higher δ_p_/δ_h_ values were considerably affected by the tail-group structure (Figure 1a and Table S3). In contrast, the radii of the spheres at low δ_p_/δ_h_ values generally increased with increasing the tail-group size (AOx8 (4.72 MPa^1/2^, 8 C’s) < AOx12 (5.35 MPa^1/2^, 12 C’s) < AOx16 (5.70 MPa^1/2^, 16 C’s)), with the exception of AOx24 (4.04 MPa^1/2^, 24 C’s). A similar trend occurred for spheres at high δ_p_/δ_h_ values (AOx8 (6.45 MPa^1/2^, 8 C’s) < AOx12 (6.84 MPa^1/2^, 12 C’s) < AOx16 (7.29 MPa^1/2^, 16 C’s) < AOx24 (8.20 MPa^1/2^, 24 C’s)).

At 3.5 wt %, the HSPs for all four compounds at low and high δ_p_/δ_h_ values were relatively similar, according to the HSPs for all compounds in the HSPiP software database (Figure 1b) [64]. In contrast, the radii of both gelation spheres were significantly affected by the tail-group structure. The radii of the spheres at lower δ_p_/δ_h_ values decreased with increasing tail-group size up to 16 C‘s (i.e., AOx8 (7.49 MPa^1/2^, 8 C’s) < AOx12 (5.46 MPa^1/2^, 12 C’s) < AOx16 (4.96 MPa^1/2^, 16 C’s)) and then increases again beyond that (i.e., AOx24 (6.00 MPa^1/2^, 24 Cs)). This could suggest that decreased hydrophobicity (or lipophilicity) favors gelator-gelator interactions over gelator-solvent interactions with low polarity solvents. For the spheres at higher δ_p_/δ_h_ values, the opposite trend is observed. The radii increased with increasing tail-group size (AOx8 (8.679 MPa^1/2^, 8 C’s) < AOx12 (9.57 MPa^1/2^, 12 C’s) < AOx16 (9.59 MPa^1/2^, 16 C’s) < AOx24 (11.70 MPa^1/2^, 24 C’s)). These results are consistent with stronger packing of longer more hydrophobic tail-groups in high polarity solvents, which would be expected thus leading to more favorable gelator-gelator over less favored gelator-high polarity solvent molecular interactions.

### 2.4. ^1^H Nuclear Magnetic Resonance (NMR) Studies 

#### Effect of Solvent 

**Figure 2:**
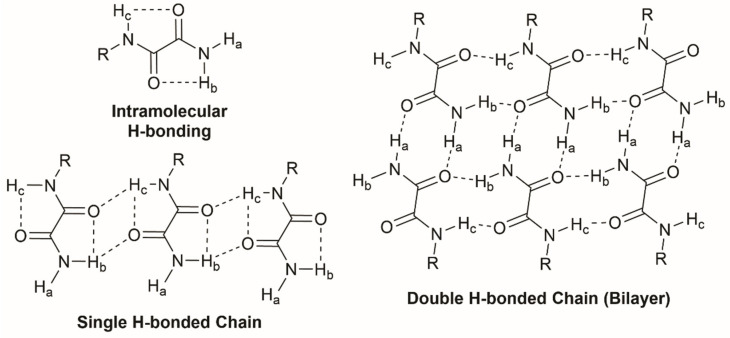
Intramolecular and proposed intermolecular H-bonding of monoalkylated oxalamides.

^1^H NMR spectroscopy was used to gain a better understanding of the driving forces for the self-assembly of the branched alkyloxalamide compounds reported here. The NH protons of oxalamide derivatives are known to form intramolecular H-bonds with the oxygen atoms of the adjacent carbonyl groups to form a planar structure via the formation of two five-member chelate rings in the monomeric state (Figure 2) [34,68,69]. For *N*, *N*′ bis-substituted oxalamides, intermolecular H-bonding via two one-point, amide-amide H-bonds can also occur to form extended H-bonded structures [70]. Monoalkylated primary oxalamides, on the other hand can form single H-bonded chains via intermolecular H-bonds, along with five-member rings formed from intramolecular H-bonds [71]. Alternatively, double H-bonded chains of bilayer aggregates via a different H-bonding pattern involving eight and ten-member H-bonded rings, where intramolecular H-bonding is absent can also form (Figure 2) [58].

First, the ^1^H NMR chemical shift dependence of all three oxalamide protons, H_a,_ H_b_ and H_c_ (Figure 2) in three different solvents at 20 mM was investigated for AOx8, AOx12, AOx16 and AOx24 (Table S4 and Figure S6). The solvents were selected to represent conditions (1) favoring gelation (i.e., toluene-*d*_8_ = tol-*d*_8_), (2) disfavoring gelation in the presence of a weak H-bond acceptor (i.e., CDCl_3_) and (3) disfavoring gelation in the presence of a strong H-bond acceptor that is known to disrupt many different H-bonding systems (i.e., DMSO-*d*_6_). For all four compounds the chemical shifts for H_a_, H_b_ and H_c_ occurred at 4.33–4.69 ppm, 6.55–6.69 ppm and 7.23–7.38 ppm in tol-*d*_8_, 5.56–5.58 ppm, 7.29–7.30 ppm and 7.30–7.37 ppm in CDCl_3_ and 7.71 ppm, 7.99 ppm and 8.52–8.54 ppm in DMSO-*d*_6_, respectively (Figures S7–S10). As expected, the chemical shifts of all three oxalamide protons were the furthest downfield in DMSO-*d*_6_, which confirms the considerable disruption of inter or intramolecular H-bonds amongst the gelator molecules at the cost of strong intermolecular H-bonds formed with the DMSO solvent. Surprisingly, the chemical shifts of the oxalamide protons H_a_ and H_b_ in CDCl_3_ are slightly more downfield shifted than in tol-*d*_8_ (Table S4), despite the fact that all four alkylated oxalamides form gels with tol-*d*_8_ and not CDCl_3_. The further downfield shift in CDCl_3_ may indicate that H_a_ and H_b_ participate more in H-bonding compared to tol-*d*_8_, but via a different H-bonding pattern that does not lead to aggregates that can form a self-assembled fibrillar network (SAFIN).

**Figure 3:**
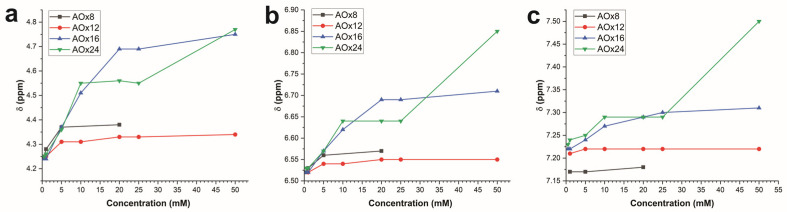
The concentration dependence of the oxalamide protons (**a**) H_a_, (**b**) H_b_ and (**c**) H_c_ in AOx8, AOx12, AOx16 and AOx24 from 0.5 to 50 mM in tol-*d*_8_.

^1^H NMR investigations in tol-*d*_8_ showed significant concentration dependence of the oxalamide N-H chemical shifts for all four oxalamides (Figure 3). Upon increasing the alkyloxalamide concentration from 0.5 to 50 mM, the maximum downfield chemical shifts (Δδ_max_) were 0.13, 0.05 and 0.01 ppm in AOx8, 0.09, 0.03 and 0.01 ppm in AOx12, 0.45, 0.17 and 0.08 ppm in AOx16, and 0.52, 0.32 and 0.27 ppm in AOx24 for H_a_, H_b_ and H_c_, respectively. The largest Δδ_max_ for H_a_ suggests that H_a_ is more sensitive to intermolecular bonding, since H_b_ and H_c_ are better spatially and geometrically preorganized for intramolecular H-bonding with the oxalamide carbonyl group oxygen lone pairs (Figure 2). Additionally, the Δδ_max_ values for AOx16 and AOx24 are similar, but significantly larger than the relatively similar values for AOx8 and AOx12, which suggests that the oxalamide protons of the gelators with larger tail-groups participate more in intermolecular H-bonding interactions and correlates well with the lower CGC values for AOx16 (0.5 wt %, 16 mM) and AOx24 (0.5 wt %, 12 mM) over AOx8 (2 wt %, 100 mM) and AOx12 (1.9 wt %, 74 mM, Table S1). Note that the general increase in Δδ_max_ (H_a_, H_b_ and H_c_) with decreasing CGC and increasing tail-group size also correlates well with the qualitative increase in solubility of AOx8 < AOx12 < AOx16 < AOx24. The order of increasing solubility is expected as larger, more lipophilic alkyl tail-groups are well-known to improve the solubility in low polarity, aprotic, lipophilic solvent such as tol-*d*_8_. 

The ^1^H NMR spectra for all four compounds were sharp at 0.5 mM in tol-*d*_8_ and became increasingly broad with increasing alkyl oxalamide concentration (Figures S7–S10). Such signal broadening at higher concentrations is consistent with the formation of increasingly large polymeric assemblies that are less mobile in solution [72,73]. The concentration at which the onset of broadening occurs increases with increasing tail-group size; i.e., AOx8 (5 mM) < AOx12 (10 mM) < AOx16 (25 mM) < AOx24 (25 mM). These results indicate that smaller, less lipophilic tail-groups result in the formation of large, less mobile aggregates at lower concentrations in a lipophilic solvent such as tol-*d*_8._ Less lipophilic tail-groups also likely promote the formation of higher order aggregates at lower concentrations due to side chain inter-aggregate packing interactions (i.e., bundling of fibers), rather than a bulky steric barrier promoting longer, thinner, individual fibers.

In the aliphatic region of the ^1^H NMR spectra, a second signal for the protons of the methylene group directly attached to the oxalamide group (i.e., NH-C*H*_2_) appears at ~3.1 ppm in addition to the original signal at ~3.0 ppm (Figures S7–S10). The concentration range at which this new signal first emerges increases with increasing tail-group size: AOx8 (<0.5 mM), AOx12 (1–5 mM), AOx16 (10–25 mM) and AOx24 (25–50 mM). This new signal increases in intensity at the expense of the original signal with further increase in concentration, which suggests the two differ by many alkyloxalamide molecules. The results also suggest that a new discrete, aggregated species (i.e., a higher order aggregate) forms in which the N-C*H*_2_ protons are situated in a less shielded environment. Perhaps extensive side chain packing interactions in a more highly aggregated state restricts rotation about the N-CH_2_ bonds so that methylene protons spend more time oriented into the diamagnetic anisotropic deshielding region of the proximal carbonyl group of the same or other neighboring molecules within the aggregate. 

### 2.5. FT-IR Spectroscopy

**Figure 4:**
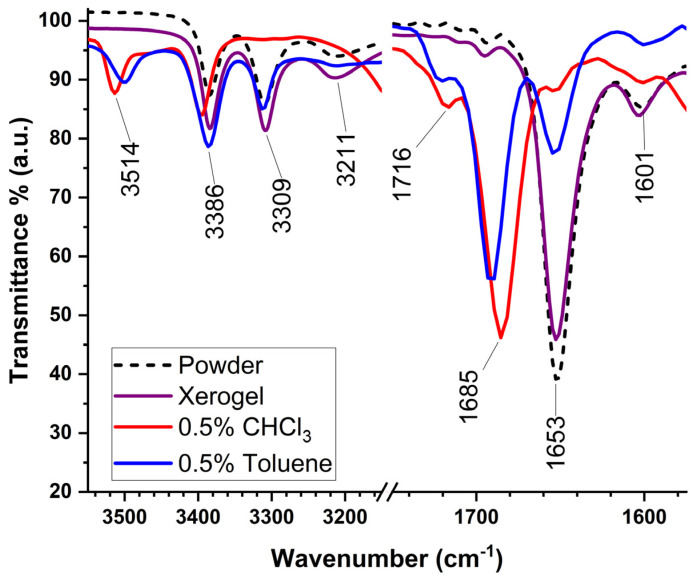
FTIR spectra of AOx24 solid, xerogel film and in chloroform and toluene solutions (0.5 wt %).

In FTIR spectra of amides and oxalamides, the N-H stretching region usually exhibits stretching vibration bands at ~3400 and ~3300 cm^−1^, due to non-H-bonded and H-bonded N-H groups, respectively [74]. These signals may be broad reflecting a distribution of H-bonded groups of varying strength dictated by distance and geometry. Additionally, the amide I band due to C=O stretching is sensitive to H-bonding. Highly ordered, strongly H-bonded carbonyl groups exhibit vibrational bands at ~1650 cm^−1^, while the vibrational bands of disordered, H-bonded carbonyl groups and free carbonyl groups are typically shifted to higher wavenumbers between ~1670 and ~1685 cm^−1^. Overall, the FTIR spectra for the branched alkyloxalamide gelators were similar for the bulk powders, xerogels, and solutions (i.e., CHCl_3_ and toluene). Representative FTIR spectra are shown in Figure 4 using the best gelator AOx24 as an example.

For AOx12, AOx16 and AOx24, at least three vibrational bands are expected in the N-H stretching region (Table S5). One signal should be present for the secondary amide group and two more for the two primary oxalamide amide N-H groups. FTIR spectra for the bulk powders, xerogels and toluene solutions of all four compounds showed two major vibrational bands at ~3380 and ~3310 and a minor band at ~3185 cm^−1^, which are all consistent with H-bonded amide N-H groups (Figure 4 and Table S5). In contrast, the FTIR spectra of CHCl_3_ solutions displayed two major vibrational bands at ~3515 and ~3390 cm^−1^ along with two minor vibrational bands at ~3450 cm^−1^ and ~3310 cm^−1^. The bands at ~3515 and ~3450 cm^−1^ are consistent with non H-bonded amide N-H groups while the bands at ~3390 and ~3310 cm^−1^ are due to H-bonded amides. Interestingly, the amide N-H stretching region of the FTIR spectrum of AOx24 in toluene (20 mM) displayed a similar pattern as the spectrum in CHCl_3_, indicating the presence of both free and H-bonded amide N-H groups.

The amide I carbonyl stretching regions of the FTIR spectra of the bulk powders of AOx12, AOx16 and AOx24, xerogels of AOx12, AOx16, AOx24, and toluene solutions of AOx12 and AOx16 were similar, displaying a minor vibrational band at ~1685 cm^−1^, which corresponds to a disordered, H-bonded carbonyl group in addition to the single major vibrational band at ~1655 cm^−1^ corresponding to an ordered H-bonded carbonyl group (Figure 4 and Table S5). In contrast, the FTIR spectra of all four compounds in CHCl_3_ show minor vibrations at ~1720 and ~1650 cm^−1^, which are consistent with free and ordered H-bonded carbonyl groups, in addition to a strong vibrational band at ~1685 cm^−1^, which is consistent with disordered, H-bonded carbonyl groups. Finally, the amide I, carbonyl stretching region for the toluene solution of AOx24 was different from AOx12 and AOx16. The spectrum for AOx24 displayed two minor bands at ~1720 and ~1650 cm^−1^, which are consistent with free and strongly H-bonded carbonyl groups, along with a major vibrational band at ~1685 cm^−1^, which is consistent with disordered H-bonded carbonyl groups.

These results suggest the following: (1) most of the oxalamides are involved in highly ordered H-bonded aggregates in the bulk powder and xerogel samples, (2) in toluene solutions, AOx12 and AOx16 molecules are also mostly tied up in highly ordered, strongly H-bonded aggregates, (3) disordered H-bonded aggregates that cannot assemble into SAFINs but can form gels are mostly present in CHCl_3_ solutions for all gelators, and (4) at 20 mM, AOx24 primarily exists in a mixture of both disordered and highly ordered H-bonded aggregates, unlike AOx12 and AOx16, which exist mostly in the latter. Finally, (5) the solution FTIR data is consistent with the gelation data for all compounds; i.e., the FTIR spectra in CHCl_3_ are consistent with weak, randomly H-bonded aggregates that cannot form SAFINs capable of forming a gel, while FTIR spectra in toluene clearly show the presence of highly ordered, strongly H-bonded species that can form SAFINs capable of gelation. Furthermore, the FTIR data for AOx24 is also consistent with the ^1^H NMR data in that both ^1^H NMR and FTIR spectra of AOx24 in CDCl_3_/CHCl_3_ and tol-*d*_8_/toluene solutions clearly show that different H-bonding arrangements are evident. These results indicate that intermolecular H-bonding between the amides protons and the amide carbonyls of these oxalamides play a dominant role in the formation of a stable organogels.

To correlate the branched, tail-group alkyl chain length with gel properties, the effect of increasing/decreasing the twin tail-group hydrocarbon chains on the gel properties such as thermal stability, gel stiffness, mechanical strength, network morphology and molecular packing was examined. For these studies, gels with two solvents were selected, which were representative from each of the two gelation spaces at low and high δ_p_/δ_h_ values (Figure 1). HD and PG were selected as the solvents for the low and high δ_p_/δ_h_ gel space, respectively. Both HD and PG have high bps, which precludes perturbations owing to solvent evaporation during measurements. The selection of PG was also based on PG being Generally Recognized As Safe (GRAS) by the U.S. Food & Drug Administration (FDA) [75]. 

### 2.6. Thermal Stability

The thermal stability of the gels were determined by measuring the gel-to-solution transition temperatures (*T*_g_’s) using the inverted vial method at both 1 and 2 wt % (Table S6). For the gels with HD, *T*_g_ values at 1 wt % were 90, 87, 87 and 68 °C for AOx8, AOx12, AOx16 and AOx24, respectively, while the *T*_g_ values at 2 wt % were 131, 109, 94 and 70 °C. These results clearly show that increasing the size of the twin tail-groups decreases *T*_g_ in the low polarity solvent HD. In contrast, the opposite trend was observed in the highly polar solvent, PG. At 1 wt % the *T*_g_ values were 57, 68 and 70 °C for AOx12, AOx16 and AOx24, respectively, while at 2 wt %, the *T*_g_ values were 73, 81 and 84 °C. Clearly, increasing twin tail-group size increases the *T*_g_ in the highly polar solvent PG. These results show that the gel thermal stability is related to the alkyl chain length in the molecular structure, which is in agreement with other reports in the literature [21,24,76].

In order to account for the opposite trends observed for the gels with HD versus PG, we suggest that upon increasing the temperature, thermal motions may enable the hydrophobic solvent HD to insert itself better amongst longer hydrophobic tail-groups, thereby facilitating disruption of the gel network. On the other hand, the more polar, hydrophilic PG solvent molecules are less likely to disrupt the packing of the hydrophobic tail-groups due to less favorable interactions between polar and low-polarity groups. Increasing the tail-group size would be expected to increase hydrophobicity, and thus stronger hydrophobic and van der Waals interactions would be expected in the presence of a highly polar, hydrophilic solvent.

### 2.7. Rheology Studies

Oscillatory rheology studies were carried out to examine the effect of the tail-group structure on the organogel viscoelastic behavior for the gels with HD and PG. First, the gels were heated to solutions and rapidly cooled to 25 °C to monitor the evolution of the elastic (*G*′) and viscous (*G*″) moduli over time at 1 Hz and 0.1 % strain in order to confirm that *G*′ and *G*″ are at equilibrium and do not significantly evolve further over time (Figures S11 and S12). The experiments clearly showed that most of the gels were fully formed after rapid cooling within 1–2 min with a few requiring close to 5 min, and that no further increase occurs between 5–30 min (Figures S11 and S12).

Next, strain sweeps were carried out at a constant frequency in order to define the linear viscoelastic region (LVR) and measure the critical (γ_c_) and cross-over (γ_x_) strain values, which identify the onset of decomposition of the gel network and the strain at which the gel is completely destroyed, resulting in a liquid. γ_c_ is typically defined as the upper limit strain value for the LVR, when *G*′ and *G*″ start to deviate from linearity, while γ_x_ is the strain at which *G*′ = *G*″ and typically indicates the gel mechanical strength [14,77]. For the HD gels at 1 wt %, γ_c_ was 0.38, 0.48, 0.88 and 1.33 % for AOx8, AOx12, AOx16 and AOx24, respectively, which indicated that the increase in the LVR correlated very well with increasing tail-group size. Additionally, γ_x_ was 25, 55, 45 and 55% for AOx8, AOx12, AOx16 and AOx24, respectively, which indicated that the gel mechanical strength increased 2-fold upon increasing the number of tail-group C’s from 8–12. Further increasing the size of the twin tail-group alkyl chains from 12–24 C’s had little impact on the gel strength. For the gels with PG, γ_c_ was 0.60, 0.60 and 1.50% for AOx12, AOx16 and AOx24, respectively, which indicates that the LVR increased only upon increasing length of the twin tail-groups from 16–24 total C’s. The γ_x_ values were 10, 100 and 100%, which indicates that the gel strength increases 10-fold upon increasing the total number of tail-group C’s from 12–16, beyond which no further increase occurred. Both trends suggest that increasing the tail-group size increases the LVR and the strains at which the gels begin to flow, which usually reflects increased cross-linking [77].

**Figure 5:**
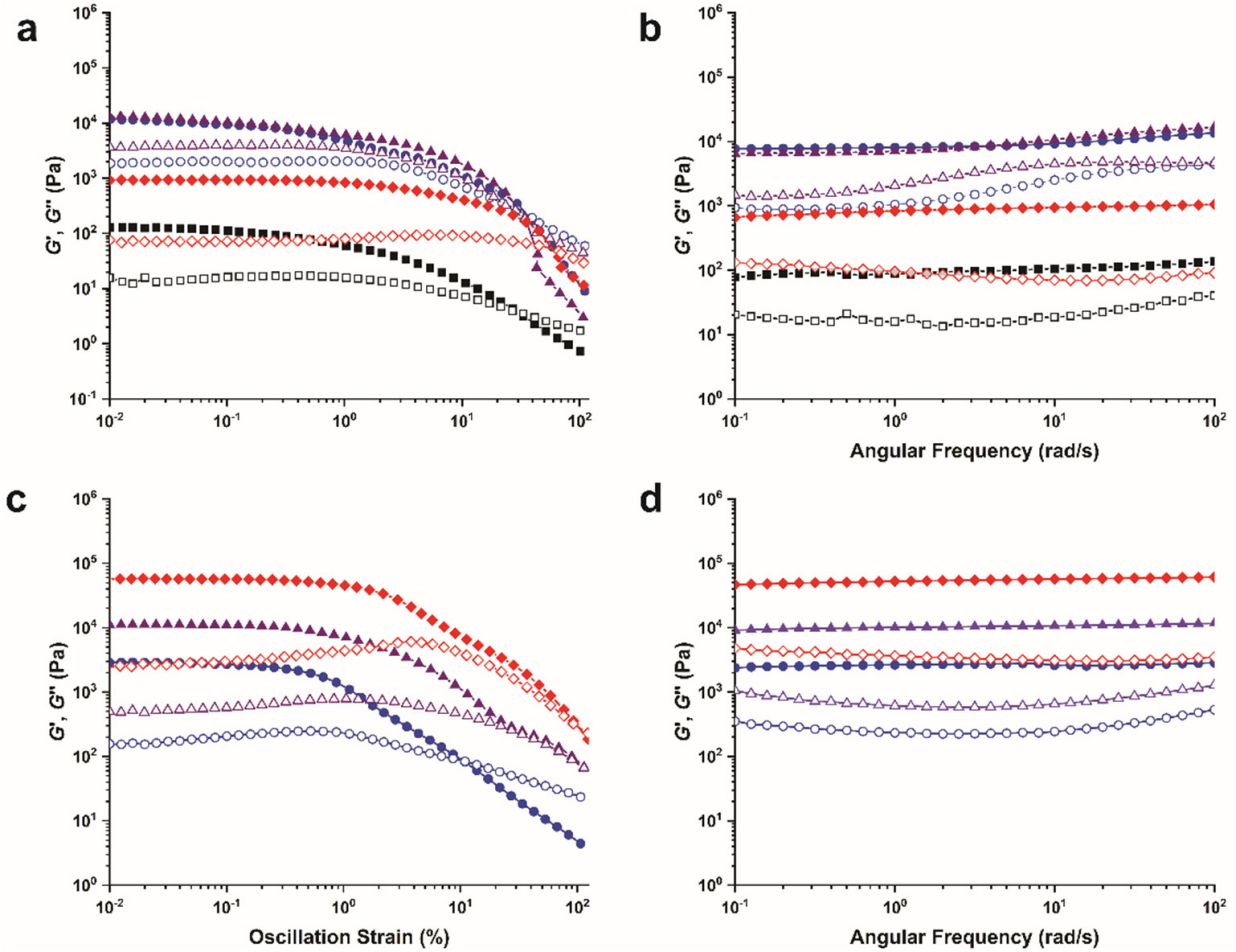
Rheology data for organogels from alkylated oxalamides. Strain (**a**) and frequency (**b**) sweeps for gels of AOx8, AOx12, AOx16, and AOx24 with hexadecane at 25 °C at 1 wt %. Strain (**c**) and frequency (**d**) sweeps for gels of AOx12, AOx16, and AOx24 with PG at 25 °C at 1 wt%. ⯀*G*′, □ *G*″ = AOx8. ● *G*′, ○ *G*″ = AOx12. ▲ *G*′, △ *G*″ = AOx16. ♦ *G*′, ♢ *G*″ = AOx24.

Frequency sweeps confirmed that all of the organogel samples exhibited true solid-like behavior since both *G*′ and *G*″ were independent of frequency and *G*′ was several times greater than *G*″ [14]. For the 1 wt % gels with HD, the *G*′ values were 105, 9230, 10,714 and 945 Pa for AOx8, AOx12, AOx16 and AOx24, respectively (Figure 5b and Table S7). These results clearly show a direct correlation between increased gel stiffness and increased number of twin tail-group C’s between eight to 16 C’s. Between 16 to 24 C’s, the gel stiffness decreased with increased C’s by an order of magnitude. In contrast, a slightly different trend was observed for the gels with PG at 1 wt %. The *G*′ values were 2600, 10,728 and 56,796 Pa, which clearly shows a direct correlation between increased gel stiffness with increased number of C’s in the twin alkyl tail-groups. Overall, the larger magnitude of γ_c_, γ_x_ and *G*′ for the gels with PG relative to HD reflect differences in fiber morphology, size and size distribution, the nature of the cross-links, cross-link density and network homogeneity, which are likely different in low versus high polarity solvents [77]. These results clearly show that differences in the molecular structures of the twin alkyl tail-groups do have a significant impact on both the mechanical stiffness and strength of the organogels, which is agreement with other reports in the literature [25,28].

Temperature sweeps were also carried out in order to gain insight into the structure of the organogels at elevated temperatures. The gels were heated to temperatures above the *T*_g_, which occurs when *G*′ = *G*″. For the gels with both solvents, *T*_g_ generally increased with increasing tail-group size. The *T*_g_ values for the gels from AOx8, AOx12, AOx16 and AOx24 with HD were 96, 110, 114 and 81 °C, respectively, while the *T*_g_ values for the gels from AOx12, AOx16 and AOx24 with PG were 66, 64 and 99 °C, respectively (Table S6 and Figures S11 and S12). As in the case with *G*′ for the gels from AOx24 with HD, *T*_g_ was also the exception, dropping from 114 to 81 °C upon increasing the tail-group size from sixteen to 24 C atoms. The overall larger *T*_g_ values for the gels with HD over PG could be attributed to the stronger ability of PG to disrupt H-bonding interactions over the low polarity, aprotic solvent HD, which cannot form strong H-bonds. 

Although the *T*_gel_ values determined using benchtop methods (i.e., falling ball, inverted vial) are convenient and do not require expensive instruments, the values are less accurate than *T*_g_ determined using other methods (i.e., NMR, DSC, rheology) [78]. Therefore, the *T*_g_ values determined by rheology here are likely closer to the actual *T*_g_ values, while the inverted vial values likely correspond to the decomposition temperatures of the organogel networks (i.e., *T*_dec_) rather than gel-to-solution transitions [79]. 

### 2.8. Polarized Optical Microscopy (POM)

**Figure 6:**
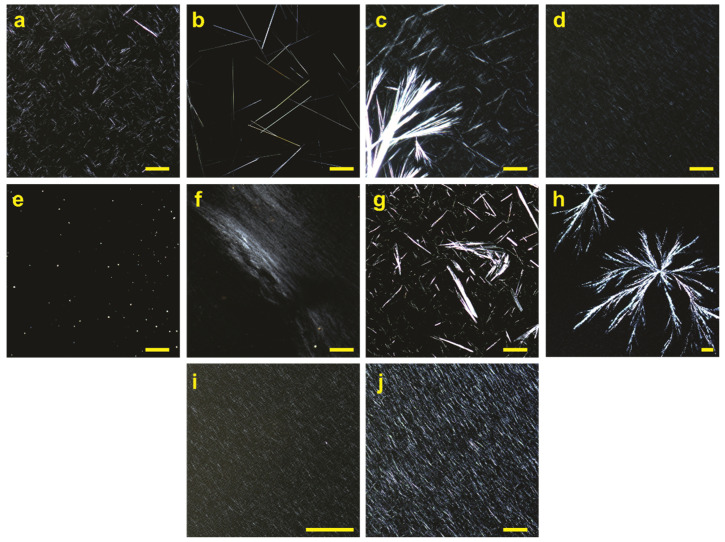
Polarized optical micrographs of gels of alkylated primary oxalamides at 1 wt % with hexadecane and propylene glycol (**f**–**i**). Hexadecane: (**a**) and (**b**) AOx8, (**c**) AOx12, (**d**) AOx16, (**e**) AOx24 and (**f**) AOx24 (after 17 h). Propylene glycol: (**g**) AOx12, (**h**) and (**i**) AOx16 and (**j**) AOx24. All images were acquired at the same magnification of 100×. The scale bar is 100 μm.

**Figure 7:**
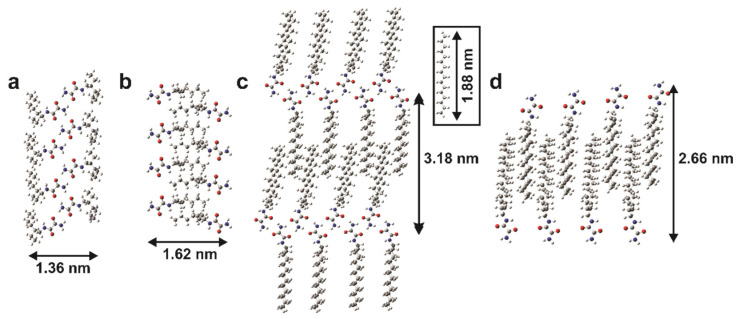
Proposed structures of the fibers that form the gel phases for AOx8 and AOx24 in HD and PG based on semi-empirical calculations (AM1) and PRXD patterns. (**a**) AOx8 in HD, (**b**) AOx8 in PG (**c**) AOx24 in HD and (**d**) AOx24 in PG. In inset a molecule of HD at the same molecular scale.

The morphology of the primary alkylated oxalamide gel networks with HD and PG were investigated by POM. The samples were prepared using conditions matching the conditions used for the samples for rheology analysis. Small amounts hot solutions (above *T*_gel_) were sandwiched between a hot glass microscope slide with cover slip and then allowed to cool to 21 °C for at least 5 min. before imaging. In general, the nanofiber width decreases with increasing tail-group size for the gels with both solvents. The POM images of the gel of AOx8 with HD exhibited biphasic networks composed of smaller and larger size distributions of highly birefringent rod-shaped structures (Figure 6a,b). In contrast, AOx12 and AOx16 exhibited more dense, moderately birefringent films (Figure 7c,d, respectively) indicating the ordered arrangements of gelator molecules in the network of fibrous aggregates. The gel from AOx24 with HD initially did not exhibit any birefringent structures, only dots (Figure 6e), which may indicate the presence of very small spherulites dispersed in a sparse fibrous network of thin fibers, below the resolution limit of POM. After 17 h, birefringence from AOx24 is stronger, exhibiting striations, which could be a network of fibrous structures (Figure 6f). While the gel from AOx8 was only stable for a few hours, the fully developed gels from AOx12, AOx16 and AOx24 with HD after one month showed highly birefringent network structures that were clearly composed of fibrous aggregates (Figure S13). POM images of the gels from AOx12, AOx16 and AOx24 with the highly polar solvent PG, showed sparse networks of straight rods (Figure 6g), mixtures of highly birefringent dendrites (spherulites) and less birefringent, dense fibrous networks (Figure 6h,i) and a highly birefringent, dense fibrous network (Figure 6j), respectively. For AOx16, the weak birefringence of the fibers (Figure 6i) may suggest that they have very small diameters in contrast to the highly birefringent fibers observed for AOx24 (Figure 6j). The above results clearly show that the molecular structure of the alkyl tail-groups has an important effect on the supramolecular structure of the organogels in both HD and PG, which is consistent with other reports in the literature [21,29,31].

The network morphologies shown in the POM images are consistent with the corresponding rheology data for the gels with both HD and PG solvents. For the gels with HD, the gel stiffness increases with tail-group size from 8 to 12–16 C’s (Figure 5 and Table S7), which correlates well with the high density SAFINs of AOx12 and AOx16 (Figure 6c,d) compared to AOx8 (Figure 6a). Stiffness then drops off above 16 C’s because the size of the fibers is reduced, since fibers of AOx24 are not visible even though a gel has formed (Figure 6e). The bulkier C24 tail-group probably offers greater steric stabilization against bundling yielding nanofibers with widths below the resolution of the optical microscope, at least at early gel times, while shorter tail-groups (i.e., 8 and 12 C’s) seem to facilitate more favorable molecular packing into larger, more crystalline fibrous aggregates. On the other hand, the critical strain and the overall mechanical strength of the gels progressively increase with increasing number of tail-group C’s, while the fibrous aggregate size (i.e., width) appears to decrease. The relatively high mechanical strength of the SAFIN from AOx24 with HD, despite the 10-fold lower stiffness, may indicate a more highly cross-linked network, although the fibrous aggregates are smaller and more easily broken, but greater in number. For the gels with PG, the POM images clearly show that the SAFIN density increases with increasing tail-group size from 12 to 24 C’s, which correlates well with the concomitant increase of the gel stiffness (*G*′), onset of breaking (i.e., γ_c_) and strain at which the gel is destroyed (i.e., γ_x_ where *G*′ = *G*″). The longer, more lipophilic tail-groups likely promote stronger intermolecular van der Waals packing and solvophobic interactions in the presence of a highly polar solvent.

### 2.9. X-ray Diffraction Study

The gels with HD and PG were examined using x-ray diffraction (XRD) in order to gain insight into the molecular packing within the gel fibers (Figures S14 and S15). We hypothesized that in the low polarity solvent HD the primary oxalamides would organize into a head-to-head lamellar organization to maximize favorable interactions with the lipophilic tail-groups and minimize unfavorable interactions with the polar head-groups. In contrast, we expected a tail-to-tail packing with the high polarity solvent PG in order to minimize unfavorable interactions with the hydrophobic tail-groups and maximize interactions with the polar head-groups. These expectations are reasonable based on: (1) the amphiphilic nature of the gelators, (2) the low polarity of HD matching the low polarity hydrocarbon tail-groups and (3) the high polarity of PG matching the polar oxalamide head-groups and capability to form H-bonding interactions between the hydroxyl groups and the oxalamide N-H and C=O groups, respectively.

The XRD pattern for the gel of AOx8 with HD exhibited the highest crystallinity as evidenced by the relative sharp reflections at 1.36, 0.85, 0.37, and 0.30 nm (Figure S14). The largest d-spacing of 1.36 nm is close to the extended length of a single molecule of AOx8 (~1.27 nm). The smaller *d*-spacings of 0.37 and 0.30 nm may be relative to the instauration of the H-bonds between the oxalamide head-groups. These results seem consistent with fibers formed of AOx8 molecules placed in a head-to-head configuration, where the width of the chain is ~1.36 nm (Figure 7a). For the gel with PG, AOx8 gave a diffraction pattern with very broad reflections (Figure S15) with respect to the gel formed in HD. Broad reflections are located at 1.62 and 1.39 nm. In this case, it is hypothesized that the fibers are formed by hydrophobic interactions of the short alkyl chains, leaving the oxalamide head-groups to freely interact in H-bonding network with the solvent (Figure 7b).

The largest *d*-spacings in the XRD patterns for AOx12, AOx16, and AOx24 in gels with HD occurred at 2.34, 2.25 and 3.18 nm, respectively, which are larger than the lengths of the extended single molecules but smaller than the corresponding extended H-bonded dimers. These results may suggest that these compounds form bilayer type aggregates with interdigitated tail-groups. For these three compounds, the lengths of the alkyl chains are long enough to fully accommodate the HD molecules within the bilayer edifice. This proposed supramolecular organization is shown in Figure 7c using AOx24 as an example, where the inset shows a molecule of HD drawn at the same scale. 

For the gels with PG, the largest *d*-spacings for AOx8, AOx12, AOx16 and AOx24 increased with increasing tail-group size (i.e., 1.62, 1.83, 2.25 and 2.66 nm, respectively, Figure S15). These values are 115–125% larger than single extended molecules, but yet are only 60–70% the width of extended dimers. These results are consistent with the formation of a tail-to-tail, inverted bilayer where the tail-groups are interdigitated (Figure 7c,d). The interlayer distance is obviously shorter than what was observed for the respective gels in HD, which is consistent with a higher degree of interdigitation and that within the bilayer edifice, no solvent molecules will be accommodated due to repulsive hydrophobic interactions with the highly polar PG molecules. The oxalamide groups may form intermolecular H-bonds to form single chains at the bilayer periphery and can also interact with the polar PG solvent as illustrated for AOx24 in (Figure 7d).

### 2.10. Cell Metabolism

**Figure 8:**
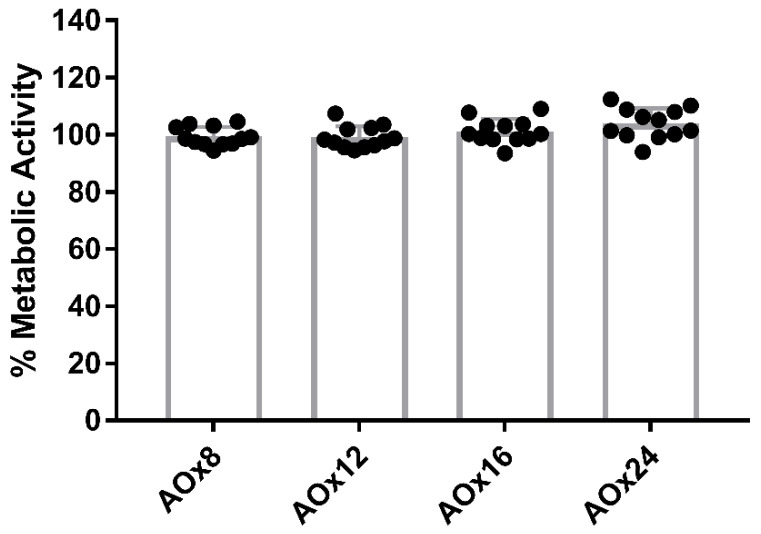
The effect of the alkyloxalamide gelators on the metabolic activity of bone marrow derived mast cells (BMMCs) at 10 μM.

Given that many oxalamide compounds are biologically and pharmaceutically active [34,35,36,37,38,39,40,41,42,43,44,45], their effect on the metabolic activity of mammalian cells was evaluated. The effect of these compounds on the metabolism of bone marrow-derived mast cells (BMMCs) was evaluated using the XTT assay which measures the nicotinamide adenine dinucleotide phosphate (NADPH)-dependent bioreductase activity of live cells, a key mitochondrial process necessary for energy generation. Mast cells are immune cells that originate from bone marrow progenitor cells before circulating in the blood steam and then migrating into the tissues where they regulate vasodilation, vascular homeostasis, immune responses, angiogenesis and venom detoxification [80,81]. As key effector cells in allergic inflammation and adverse drug reactions, mast cells are frequently activated in patients that have hypersensitivity reactions to medications or implant materials [82,83,84]. The BMMCs used in this study were cultured from wild type mice and do not harbor any known mutations, displaying normal senescent properties consistent with healthy primary cells. At 10 μM, all four primary oxalamides had no effect on the metabolic activity of BMMCs after 24 h exposure (Figure 8), which suggests that these compounds are not cytotoxic and are unlikely to affect the viability of these cells. The compounds should be suitable for biomedical applications, at least in terms of their effect on these immune cells. To the best of our knowledge, this is the first study to examine the effect of a LMWG on mast cell metabolic activity.

## 3. Conclusions

In summary, a series of primary mono-*N*-alkylated oxalamide derivatives, which have similar molecular structures but different twin alkyl tail-group chain lengths of 6/2 (8), 8/4 (12), 10/6 (16) and 14/10 (24) C atoms attached to one of the oxalamide nitrogen atoms. The gelation behaviors of these compounds was examined in 31 different solvents with different properties such as polarity and H-bonding ability. All four compounds formed stable organogels with mostly the same solvents, both high and low polarity, with a few exceptions. While the molecular structure of the tail-groups did not have a significant effect on the gelation behavior, the CGC did generally decrease with increasing sizes of the twin tail-groups. ^1^H NMR and FTIR studies confirmed that H-bonding interactions are an important driving force in the self-assembly and gelation process. Vial inversion, rheology and microscopy studies also confirmed that the molecular structure of the twin tail-groups have a significant effect on the organogel thermal stability, stiffness, strength, and aggregate homogeneity and network morphology. The results clearly showed that the measured properties did correlate well with increasing/decreasing size of the twin tail-groups, and that the direction and magnitude is dependent on the type of solvent. PXRD results were consistent with the lamellar packing of the primary mono-*N*-alkylated oxalamide derivatives. For the gels with the low polarity solvent HD, the layer *d*-spacing values were consistent with the molecular lengths and a gel architecture involving a head-to-head packing arrangement governed by the instauration of an H-bonded network between the polar oxalamide head-groups. On the other hand, for the gels with the highly polar, protic solvent PG, the layer *d*-spacing values were also consistent with the molecular lengths and a gel architecture, but directed by hydrophobic interactions between the alkyl chains via an interdigitated tail-to-tail packing arrangement. This work expands upon the development of gelators based on the oxalamide group and should lead to generation of new functional, soft nanomaterials for different applications in the future. Since all four oxalamides did not present any cytotoxic effect, we are currently developing such materials for applications such as drug delivery and tissue engineering, which will be reported in due course.

## 4. Materials and Methods

### 4.1. Materials

All chemicals were commercially available and used without further purification unless stated otherwise. ISOCARB 12 (2-butyloctanoic acid), ISOCARB 16 (2-hexyldecanoic acid), and ISOCARB 24 (2-decyltetradecanoic acid) were acquired from Sasol America (Houston, TX, USA). Capmul MCM C8 (monodiglycerides of caprylic acid) was used as received from the Abitec corporation (Janesville, WI, USA). Hexadecane (>98%) was acquired from TCI America (Portland, OR, USA). CDCl_3_ (D, 99.8%), DMSO-*d*_6_ (D, 99%), acetone-*d*_6_ (D, 99.9%) and toluene-*d*_8_ (D, 99.5%) were used as received from Cambridge Isotope Laboratories (Tewksbury, MA, USA). Benzyl alcohol (99.8%), chloroform (99.8%), cinnamaldehyde (95%), corn oil, diethyl ether (99%), dimethyl sulfoxide (99.9%), 1,4-dioxane (99.5%), dodecane (≥99%), ethanolamine (≥98%), 2-ethyl-1-hexylamine (98%), isopropyl myristate (USP/FCC), lithium aluminum hydride (95%), methyl ethyl ketone (≥99%), oleic acid (90%), oxalyl chloride (≥99%), propylene glycol (≥99.5%), propylene carbonate (99.7%), sesame oil, soybean oil, sodium hydroxide (99%), sodium chloride (>99%), triethylamine (≥99.5%), β-mercaptoethanol and Roche Cell Proliferation Kit II (XTT = 2,3-bis-(2-methoxy-4-nitro-5-sulfophenyl)-2H-tetrazolium-5-carboxanilide) were acquired from the Sigma-Aldrich chemical company (Mississauga, ON, Canada). Acetone (Acros, 99.9%), acetonitrile (99.9%), ammonium hydroxide solution (JT Baker, 10–35% NH_3_ basis), 1-butanol (99.9%), castor oil, dichloromethane (99.8%), ethyl chloro oxoacetate (98%), cyclohexane (99.8%), ethyl acetate (99.5%), hexanes (98.5%), *N,N*-dimethylformamide (Acros, anhydrous, >99.8%), lactic acid, methanol (99.8%), olive oil, sodium sulfate (99%), tetrahydrofuran (99.8%), toluene (99.9%), xylenes (99.9%), 10% heat inactivated fetal bovine serum (FBS), sodium pyruvate, and non-essential amino acids were acquired from Fisher Scientific Canada (Toronto, ON, Canada). Penicillin (100 U/mL)/streptomycin (100 µg/mL), 4-(2-hydroxyethyl)-1-piperazineethanesulfonic acid (HEPES) and Murine interleukin-3 (IL-3) were obtained from Peprotech (Cranbury, NJ, USA). Roswell Park Memorial Institute (RPMI) media was acquired from Gibco (Waltham, MA, USA). Bovine serum albumin (BSA), phosphate-buffered saline (PBS) pH 7.4, anti-mouse FcεRI-allophycocyanin antibody, anti-mouse CD117-phycoerythrin (PE) antibodies, IgG-PE, Armenian hamster IgG-PE were all purchased from eBiosciences (San Diego, CA, USA). Deionized (DI) water was purified using a Milli-Q ultrapure unit. Anhydrous THF and dichloromethane solvents were obtained using an MBraun solvent purification system (SP05-172, Stratham, NH, USA).

### 4.2. Nuclear Magnetic Resonance (NMR) Spectroscopy

^1^H and ^13^C NMR spectra were recorded with a Varian (Palo Alto, CA, USA) AMX600 spectrometer. Chemical shifts (*δ*) are in ppm. Multiplicities are denoted as follows: s = singlet, d = doublet, t = triplet, m = multiplet, br = broad. For the concentration dependent studies in toluene-*d*_8_, solutions of AOx8 (0.5–20 mM) and AOx12, AOx16 and AOx24 (0.5–50 mM) were prepared by dissolving the corresponding alkyloxalamide bulk powders in toluene-*d*_8_ at 50 mM in an ultrasonic bath (Bransonic 3510R-MT, 117 V, 50–60 Hz). The samples were not heated in order to avoid gelation. Serial dilutions were carried out to give solutions at lower concentrations. The compound AOx8 formed slightly turbid, homogeneous sols between 0.5–20 mM. The compound AOx12 formed clear solutions between 0.5–10 mM and turbid sols at 25 and 50 mM. The compounds AOx16 and AOx24 gave clear solutions between 0.5–25 mM. At 50 mM compound AOx16 formed a turbid gel while AOx24 gave a clear gel.

### 4.3. Mass Spectrometry (MS)

High resolution electrospray ionization (HR-ESI) mass spectra were acquired using an Agilent Technologies (Santa Clara, CA, USA) 6220 orthogonal acceleration time-of-flight (oaTOF) instrument in positive ion mode.

### 4.4. Gelation Tests

The gelation tests were carried out using the ‘‘vial inversion test.” Gelator powder (1–35 mg) and solvent (1 mL) were placed into a glass vial (4 mL, outer diameter = 15 mm, height = 45 mm) with a threaded top, which was sealed with Teflon-tape and a screw-cap lid. The mixture was then sonicated in a Bransonic 3510R-MT ultrasonic bath (117 V, 50–60 Hz, Branson Ultrasonics, Brookfield, CT, USA) for times ranging between 5 s and 2 min. until a fine suspension was obtained. The mixture was then heated using a heat gun until the solid completely dissolved and a clear solution was obtained. Gels were formed by allowing the solutions to slowly cool to room temperature for at least 30 min. The gels were denoted as clear (CG), turbid (TG), and opaque (OG), (I) for mixtures that were not soluble with heat, (S) for mixtures that were soluble after heat and remained soluble after cooling at room temperature, and (P) for mixtures that were soluble after heating but precipitated after cooling at room temperature. The critical gel concentration (CGC) is the minimum gelator concentration that formed stable gel that did not fall upon inverting the vial.

### 4.5. Hansen Solubility Parameter Analysis

The gelation spheres from the gelation tests for each gelator at 1 wt % and 3.5 wt % were determined using the Genetic Algorithm, Double Sphere fitting procedure in the HSPiP 5.4.07 x64 software. Since this procedure does not give a unique answer, the coordinates of the centers (*δ*_p,_ *δ*_h_, 2*δ*_d_) and radii (in MPa^1/2^) of the final gel spheres were taken as the average of ten calculations for each gelator at each concentration.

### 4.6. Fourier Transform Infra-Red (FT-IR) Spectroscopy 

FT-IR measurements on solid and gel samples were carried out using a Harrick MVP-pro Attenuated Total Reflectance (ATR) accessory (Harrick Scientific Products, Inc., Pleasantview, NY, USA). All FTIR spectra were recorded at 23 °C using a Digilab (Hopkinton, MA, USA) FTS 7000 Series spectrometer. Solution measurements were carried out using a Thermo Spectra Tech FT04-035 Demountable Pathlength CaF_2_ Cell (P/N 700-0085) with a 0.1 mm path length.

### 4.7. Inverted Vial Gel Melting Experiments 

The gel-to-solution transition temperatures (*T*_gel_) also were also determined using the “inverted vial” method. Vials containing the gels at different gelator concentrations sealed with screw caps and Teflon tape were inverted and heated at a rate of 1–2 °C/min. *T*_gel_ was accepted to be the temperature at which the gel had completely fallen to the bottom of the vial.

### 4.8. Rheology Measurements

Rheological measurements were carried out with a Discovery HR-30 rheometer (TA instruments, New Castle, DE, USA). Oscillatory viscoelastic measurements were carried out using a parallel plate geometry and a Peltier system for temperature control to measure the storage or elastic modulus (*G*′) and the loss or viscous modulus (*G*″). A 40 mm diameter cross-hatched plate was used with a 500 μm gap. The HD gel samples were formed in situ directly on the Peltier plate, while the PG gels were formed ex situ in a glass vial and were transferred to the Peltier plate by scooping out with a spatula. A typical procedure for the gels was as follows. Gel samples were placed on the Peltier plate and heated to above the gel melting temperature to form a solution. The samples were then rapidly cooled to 25 °C between the plates and *G*′ and *G*″ were recorded over time until both were at equilibrium at a strain amplitude of 0.15% and a frequency of 1 Hz. Typically, *G*′ and *G*″ were at equilibrium after 5 min. Strain sweep measurements were performed between 0.01–100% at 1 Hz at 25 °C to determine the mechanical strengths of the gel samples and to determine the linear viscoelastic region (LVR). Frequency sweep measurements were then carried out using a constant strain amplitude of 0.15% between 1–600 rad/s at 25 °C. Temperature sweep measurements were recorded at a constant strain amplitude of 0.15% at 1 Hz using a heating rate of 5 °C/min. The temperature at which *G*′ = *G*″ was taken to be the gel-to-solution transition temperature (*T*_gel_).

### 4.9. Polarized Optical Microscopy (POM)

Polarized optical microscope (POM) images were acquired using a Zeiss (Toronto, ON, Canada) Axio Scope A1 in different contrast and polarization modes. Gel samples were analyzed on glass microscope slides under a glass coverslip.

### 4.10. X-ray Diffraction (XRD)

XRD data was acquired between 1° ≤ 2 Theta ≤ 30° (step size = 0.01°) with a Bruker (Milton, ON, Canada) model D8/Discover X-ray diffractometer using CuKa radiation (λ = 1.5406 Å) at 50 kV and 10 mA equipped with a Vantec500 detector. The resulting data was analysed using EVA^tm^ software.

### 4.11. Computer Modelling

The geometries of cyclic hexamers were optimized in the gas phase using the Polak–Ribiere conjugate gradient algorithm of the Hyperchem 7.51 program (Hyperchem, Hypercube Inc., Gainsville, FL, USA) through semi-empirical calculations, using the AM1 method with an RMS gradient of 0.01 kcal/Å mol.

### 4.12. Cell Culture, Flow Cytometric Analysis and Cytotoxicity Studies

BMMC cells were cultured from progenitor stem cells isolated from the femur of 10–12 week-old C57BL/6 mice in accordance to the Canadian Council on Animal Care Guidelines and the University of Alberta Animal Care Committee standard operating procedures (REO#2021.026Kulka under category “A”). The cells were cultured in RPMI media supplemented with 10% heat inactivated fetal bovine serum, L-glutamine (4 mM), β-mercaptoethanol (50 μM), sodium pyruvate (1 mM), non-essential amino acids (100 μM), penicillin (100 U/mL)/streptomycin (100 µg/mL), HEPES (10 mM) and murine IL-3 (30 mg/mL in 5% CO_2_ at 37 °C for four weeks. Prior to use, >99% of the cells expressed the mature mast cell phenotype as confirmed by flow cytometry analysis of FcεRI, the high-affinity receptor for the fragment crystallisable region (Fc region) of immunoglobulin E (IgE) and cluster of differentiation 117 (CD117) surface expression. Briefly, 2.5 × 10^5^ cells were washed in 0.1% BSA in PBS pH 7.4, blocked in 3% BSA in PBS pH 7.4 for 10 min. on ice, labelled in 3% BSA in PBS at pH 7.4 with 0.05 µg/mL anti-mouse FcεRI-APC and anti-mouse CD117-PE antibodies or their corresponding isotype controls, rat IgG-PE or Armenian hamster IgG-PE. After 30 min., the cells were then washed in 0.1% BSA in PBS pH 7.4 and analysed via flow cytometry using the Fortessa X-20 instrument from (BD Biosciences Franklin Lakes, NJ, USA).

BMMCs were treated with each alkyloxalamide and the effect on the metabolic activity was examined using the XTT Assay according to the manufacturer’s instructions. First, 10 mM stock solutions or suspensions of each alkyloxalamide in DMSO (10 μL) were added to RPMI culture media (0.990 mL) to give 100 µM stocks. Next, each 100 µM alkyloxalamide stock (10 μL) and 5 × 10^5^ cells/mL (90 μL) were mixed in a 96-well plate and incubated at 37 °C and 5% CO_2_. After 24 h, the XTT reagent (50 μL) was added and the cells were incubated further at 37 °C and 5% CO_2_. After 6 h, the absorbance was measured at 450 nm against a reference wavelength at 650 nm using a microplate reader (Varioskan Lux, Thermofisher, Waltham, MA, USA). The metabolic activity was calculated using the formula:

% Metabolic activity = (A_450_ − A_650_)_treated cells_/(A_450_ − A_650_)_untreated cells_ × 100%

The control experiment was also carried out in the same way in the absence of oxalamides. All experiments were carried out in quadruplicate.

## Data Availability

Not applicable.

## Supplementary Materials

The following supporting information can be downloaded at: https://www.mdpi.com/article/10.3390/gels9010005/s1, Figure S1a: ^1^H and ^13^C NMR spectra of AOx8, Figure S1b: High resolution mass spectrum of AOx8, Figure S2a: ^1^H and ^13^C NMR of AOx12, Figure S2b: High resolution mass spectrum of AOx12, Figure S3a: ^1^H and ^13^C NMR of AOx16, Figure S3b: High resolution mass spectrum of AOx16, Figure S4a: ^1^H and ^13^C NMR of AOx24, Figure S4b: High resolution mass spectrum of AOx24, Table S1: Gelator test data for alkylated oxalamides. Table S2: Hansen Solubility Parameters (HSPs) and outcomes of solubility/gelation tests with alkylated oxalamides. Figure S5: 3D Hansen space for gelation spheres (green) for alkyloxalamido gelators, Table S3: Coordinates of the centers of the solution (S) and gelation spheres (G1, G2) in Hansen space and their radii, Figure S6: ^1^H NMR spectra (600 MHz) of the amide region for AOx24 in different solvents (20 mM, 0.85 wt %), Table S4: ^1^H NMR chemical shifts (ppm) of H_a_ and H_b_ for alkyloxalamides (20 mM) in CDCl_3_, DMSO-*d*_6_ and toluene-*d*_8_ at 23 °C, Figure S7: ^1^H NMR (600 MHz) spectra of AOx8 in toluene-*d*_8_ from 0.5-50 mM, Figure S8: 1H NMR (600 MHz) spectra of AOx12 in toluene-*d*_8_ from 1-50 mM, Figure S9: 1H NMR (600 MHz) spectra of AOx16 in toluene-*d*_8_ from 0.5-50 mM, Figure S10: 1H NMR (600 MHz) spectra of AOx24 in toluene-*d*_8_ from 0.5-50 mM, Table S5: FTIR data for alkylated oxalamide gelators, Table S6: Gel melting point T_gel_ of gelators in hexadecane and propylene glycol at 1 and 2 wt % from inverted vial and rheology temperature sweep experiments, Figure S11: Rheology gel time and temperature sweep experiments for organogels of AOx8 (a, b), AOx12 (c, d), AOx16 (e, f) and AOx24 (g, h) with hexadecane, Figure S12: Rheology gel time and temperature sweep experiments organogels of AOx12 (a, b), AOx16 (c, d) and AOx24 (e, f) with propylene glycol, Table S7: Rheological properties of organogels with hexadecane (HD) and propylene glycol (PG) at 1 wt % at 25 °C, Figure S13: Polarized optical micrographs of gels from alkyloxalamides with hexadecane at 1 wt % after 1 month. (a) and (b) AOx12. (c) AOx16. (d) and (e) AOx24, Figure S14: Powder X-Ray diffraction patterns of organogels of alkyloxalamide gelators with hexadecane, Figure S15: Powder X-Ray diffraction patterns of organogels of alkyloxalamide gelators with propylene glycol.
