# Supplementary material for: The Effect of Branched Alkyl Chain Length on the Properties of Supramolecular Organogels from Mono-N-Alkylated Primary Oxalamides
Source: Gels. 2022 Dec 22;9(1):5. doi: 10.3390/gels9010005 (PMC9858617; doi:10.3390/gels9010005)
Supplement: Supplementary file 1 [file gels-09-00005-s001.zip › gels-2036504-supplementary.pdf]

gels

MDPI

Article

The Effect of Branched Alkyl Chain Length on the Properties of Supramolecular Organogels from Mono-*N*-Alkylated Primary Oxalamides

Khalid Azyat [1], Darren Makeiff [1,*], Bradley Smith [1], Mickie Wiebe [1], Steve Launspach [1], Ashley Wagner [1], Marianna Kulka [1] and Nicolas Godbert [2]

[1] Nanotechnology Research Center, National Research Council of Canada, 11421 Saskatchewan Drive, Edmonton, AB T6G 2M9, Canada
[2] Dipartimento di Chimica e Tecnologie Chimiche, Università della Calabria, 87036 Arcavacata di Rende, CS, Italy
* Correspondence: darren.makeiff@nrc.ca

Supplementary Materials:

Contents

Gelator Synthesis 2

Synthesis of AOx8 2

Figure S1a. ^1H and ^{13}C NMR spectra of **AOx8.** 4

Figure S1b. High resolution mass spectrum of **AOx8.** 5

Synthesis of AOx12 5

Figure S2a. ^1H and ^{13}C NMR of **AOx12** 7

Figure S2b. High resolution mass spectrum of **AOx12** 8

Synthesis of AOx16 8

Figure S3a. ^1H and ^{13}C NMR of **AOx16** 10

Figure S3b. High resolution mass spectrum of **AOx16** 11

Synthesis of AOx24 11

Figure S4a. ^1H and ^{13}C NMR of **AOx24** 14

Figure S4b. High resolution mass spectrum of **AOx24** 15

Additional Data 15

Table S1. Gelator test data for alkylated oxalamides 15

Table S2. Hansen Solubility Parameters (HSPs) and outcomes of solubility/gelation tests with alkylated oxalamides. 16

Figure S5. 3D Hansen space for gelation spheres (green) for alkyloxalamido gelators. 17

Table S3. Coordinates of the Centers of the Solution (S) and Gelation Spheres (G1, G2) in Hansen Space and Their Radii. 17

Figure S6. ^1H NMR spectra (600 MHz) of the amide region for **AOx24** in different solvents (20 mM, 0.85 wt %). 18

Table S4. ^{1}H NMR chemical shifts (ppm) of H_a and H_b for alkyloxalamides (20 mM) in $CDCl_3$, DMSO-d_6 and toluene-d_8 at 23°C. 18

Figure S7. ^{1}H NMR (600 MHz) spectra of **AOx8** in toluene-d_8 from 0.5-50 mM. 19

Figure S8. ^{1}H NMR (600 MHz) spectra of **AOx12** in toluene-d_8 from 1-50 mM. 20

Figure S9. ^{1}H NMR (600 MHz) spectra of **AOx16** in toluene-d_8 from 0.5-50 mM. 21

Figure S10. ^{1}H NMR (600 MHz) spectra of **AOx24** in toluene-d_8 from 0.5-50 mM. 22

Table S5. FTIR data for alkylated oxalamide gelators. 22

Table S6. Gel melting point T_{gel} of gelators in hexadecane and propylene glycol at 1 and 2 wt % from inverted vial and rheology temperature sweep experiments 23

Figure S11. Rheology gel time and temperature sweep experiments for organogels of 24

Figure S12. Rheology gel time and temperature sweep experiments organogels of **AOx12** (a, b), **AOx16** (c, d) and **AOx24** (e, f) with propylene glycol. 25

Table S7. Rheological properties of organogels with hexadecane (HD) and propylene glycol (PG) at 1 wt % at 25°C. 25

Figure S13. Polarized optical micrographs of gels from alkyloxalamides with hexadecane at 1 wt % after 1 month. (a) and (b) **AOx12**. (c) **AOx16**. (d) and (e) **AOx24**. The images are 1.42 µm wide. 26

Figure S14. Powder X-Ray diffraction patterns of organogels of alkyloxalamide gelators with hexadecane. 27

Figure S15. Powder X-Ray diffraction patterns of organogels of alkyloxalamide gelators with propylene glycol. 28

Gelator Synthesis

Synthesis of AOx8

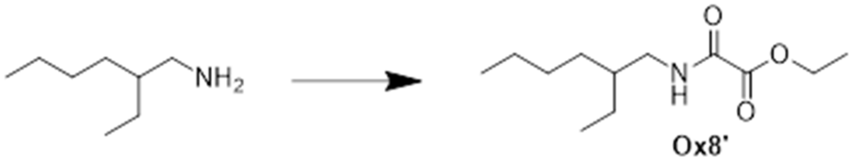

Ox8'

Ethyl chlorooxoacetate (5.28 g, 38.69 mmol) was added to a solution of 2-ethyl-1-hexylamine (5 g, 38.69 mmol) in anhydrous DCM (200 mL) at 0°C. Et_3N (7.83 g, 77.38 mmol) was then added and the mixture was slowly warmed to 23°C. After 3 h, the reaction was washed with water (2 × 25 mL) followed by brine (25 mL). The organic layer was the dried over Na_2SO_4, filtered, and the solvent removed by rotary evaporation to afford **Ox8′** as a yellow oil (8.85 g, 99%).

^{1}H NMR ($CDCl_3$, 600 MHz) 7.05 (br s, 1H), 4.30 (q, 2H, *J* = 7.2 Hz), 3.24 (m, 2H), 1.47 (m, 1H), 1.27-1.35 (m, 12H), 0.83-0.87 (m, 6H).

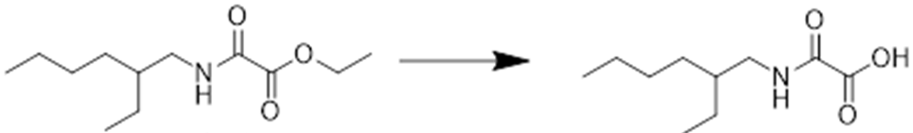

Ox8' **Ox8**

Aqueous 2.5 M NaOH (31 mL, 77.18 mmol) was added to a solution of **Ox8′** (8.80 g, 38.37 mmol) in THF (200 mL). The reaction was then stirred at 23°C for 4 h before 2M HCl was added to lower the pH to ~3. The excess THF was then removed by rotary evaporation and the aqueous mixture was then extracted with DCM (3×50 mL). The DCM extracts were combined and dried over Na_2SO_4, filtered and concentrated by rotary evaporation to afford **Ox8** as a white solid (7.70 g, 99%).

^{1}H NMR ($CDCl_3$, 600 MHz) 7.26 (br s, 1H), 3.30 (m, 2H), 1.57 (m, 1H), 1.26-1.32 (m, 9H), 0.88-0.91 (m, 6H).

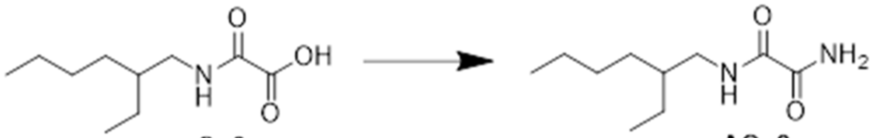

Ox8 **AOx8**

Oxalyl chloride (3.30 mL, 69.34 mmol) was added dropwise to a solution of **Ox8** (7.70 g, 38.25 mmol) in anhydrous THF (150 mL) and anhydrous DMF (3 drops) at 0°C. The reaction was then slowly warmed to 23°C. After 4 h, the excess THF was removed by rotary evaporation and the crude residue was dried *in vacuo*. Fresh anhydrous THF (200 mL) was then added and the mixture was cooled to 0°C before adding aqueous NH_4OH (10~30 %, 40 mL) dropwise. The mixture was then warmed to 23°C and allowed to stir for an additional 30 min., before the excess THF was removed by rotary evaporation. The white solid residue was dispersed in deionized water, filtered, and washed with deionized water before drying *in vacuo* for 18 h to afford the compound **AOx8** as a white solid (5.60 g, 73%).

^{1}H NMR ($CDCl_3$, 600 MHz) δ 7.38 (br s, 1H), 7.31 (br s, 1H), 5.60 (br s, 1H), 3.25 (m, 2H), 1.51 (m, 1H), 1.28-1.35 (m, 8H), 0.88-0.95 (m, 6H); ^{13}C NMR ($CDCl_3$, 600 MHz) δ 162.1, 159.4, 42.6, 39.3, 39.2, 30.9 x2, 28.8, 24.1, 22.9, 14.0, 10.8. HRMS (ESI, M^+) cald for $C_{10}H_{20}N_2O_2$ 223.1417 $(M+Na)^+$, 223.1415 $(M+Na)^+$ found.

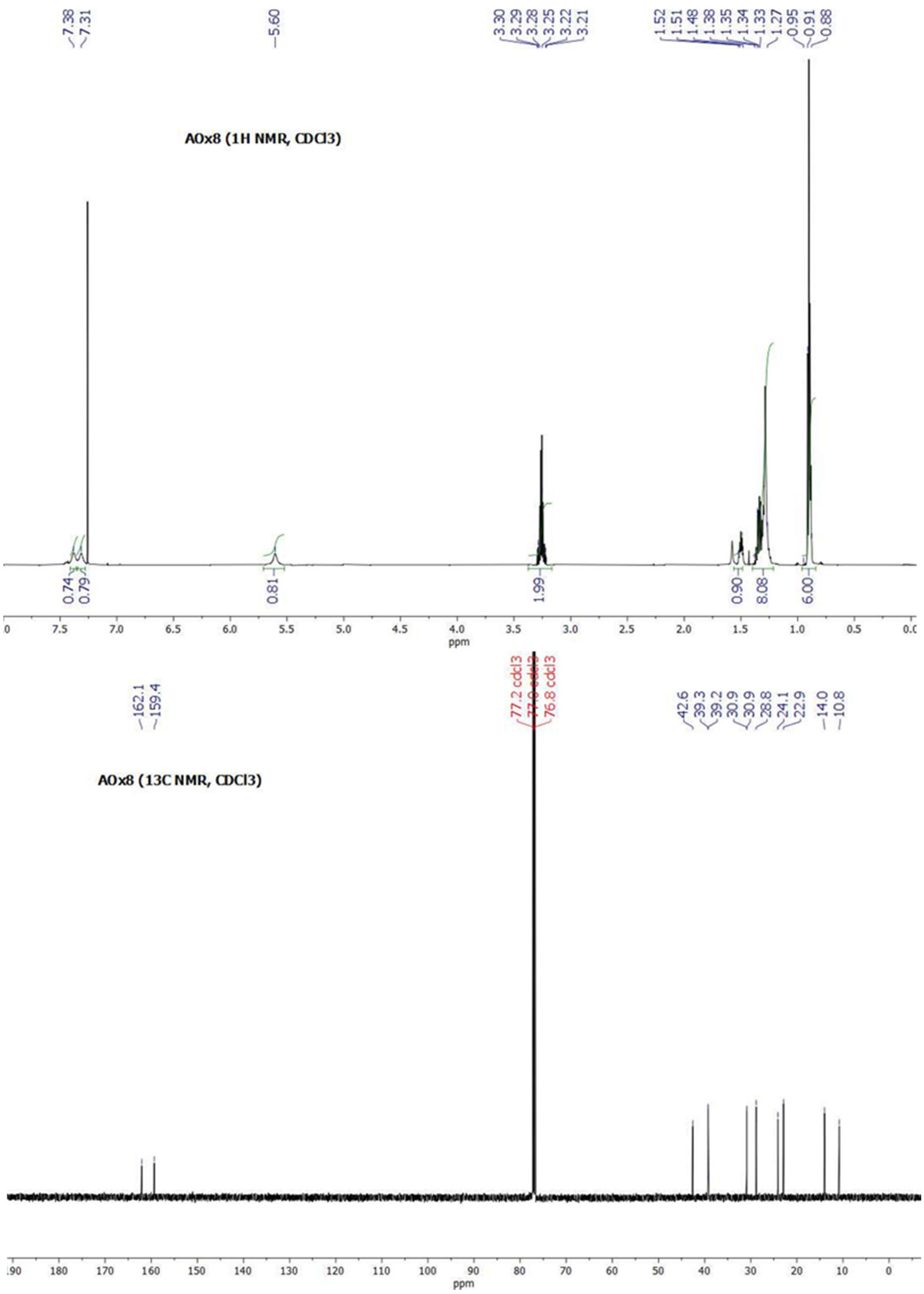

Figure S1. a. ^{1}H and ^{13}C NMR spectra of **AOx8.**

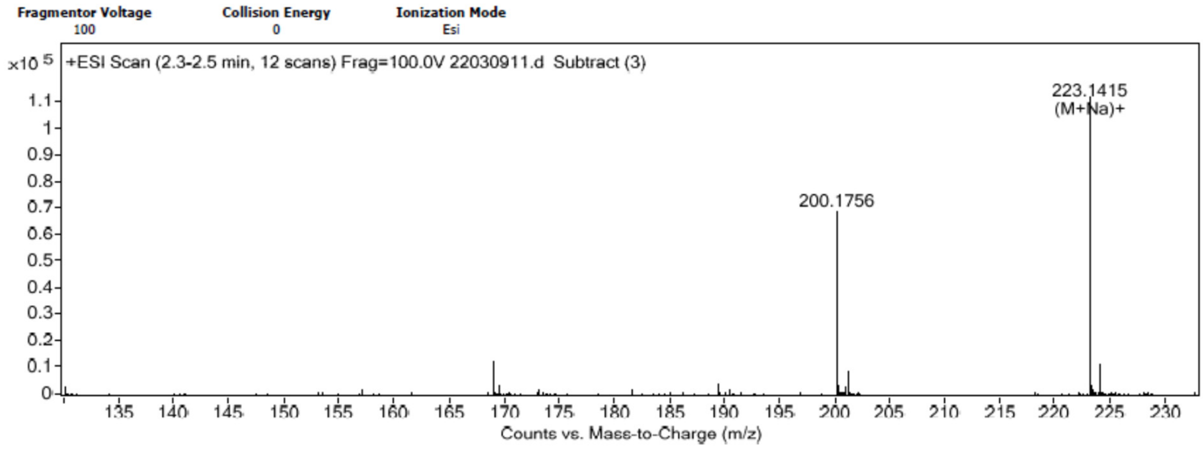

Formula Calculator Results

Formula	Ion Formula	Mass	Calc Mass	m/z	Calc. m/z	Diff (mDa)	Diff (ppm)	DBE	Ion Species	Score
C10 H20 N2 O2	C10 H20 N2 Na O2	200.1523	200.1525	223.1415	223.1417	0.22	0.97	2	(M+Na)+	83.43

Figure S1. b. High resolution mass spectrum of **AOx8.**

Synthesis of AOx12

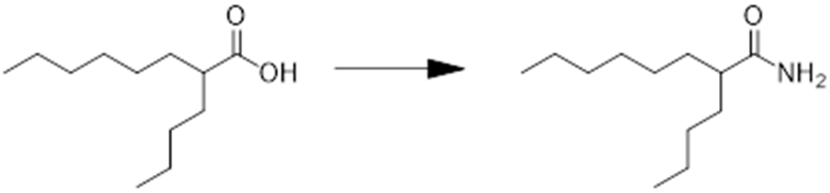

Oxalyl chloride (9.5 g, 75 mmol) was added dropwise to a solution of 2-butyloctanoic acid (10 g, 50 mmol) and anhydrous DMF (0.1 mL) in anhydrous THF (250 mL) at 0 °C. The mixture was then allowed to warm to 23 °C. After 5 h, the excess THF was removed by rotary evaporation and the crude was dried *in vacuo*. Fresh THF (200 mL) was added and the mixture was cooled to 0°C before adding aqueous NH_4OH solution (10-30 %, 40 mL) dropwise. The mixture was warmed to 23 °C. After 30 min, the excess THF was removed by rotary evaporation and the resulting white solid was filtered and washed with water and dried *in vacuo* for 18 h to give 2-butyloctanamide as a white solid (9.92 g, 99%).

^{1}H NMR ($CDCl_3$, 600 MHz) 5.40-5.60 (br m, 2H), 2.08 (m, 1H), 5.6 (br s, 1H), 1.57 (m, 2H), 1.40-145 (m, 2H), 1.26-1.32 (m, 12H), 0.85-0.89 (m, 6H).

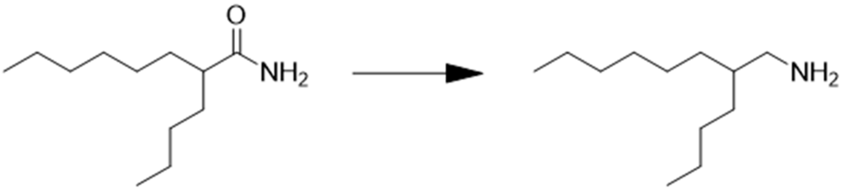

LiAlH4 (5.42 g, 142.95 mmol) was added in portions over 1 h to a solution of 2-butyloctanoic amide (9.5 g, 51.25 mmol) in anhydrous THF (250 mL) at 0°C. After 1h, the reaction was warmed to 23°C for 2 d and then refluxed for 24 h. The mixture was cooled to 0 °C and water (5.5 mL) was added dropwise over 45 min, followed by a solution of aqueous 0.63 M NaOH solution (22 mL, 13.8 mmol). After stirring at 23°C for 2 h, the mixture was filtered through celite and the excess THF was removed by rotary evaporation. The resulting aqueous phase was then extracted with DCM (200 mL). The organic layer was separated and washed with water (2×20 mL) and brine (50 mL) before drying over Na_2SO_4, filtering, concentrating by rotary evaporation and drying *in vacuo* to afford 2-butyloctylamine as a viscous yellow liquid (8.00 g, 89%).

^{1}H NMR ($CDCl_3$, 600 MHz) 2.58 (d, 1H, *J* = 5.4 Hz), 1.25-1.28 (m, 17H), 0.85-0.89 (m, 6H).

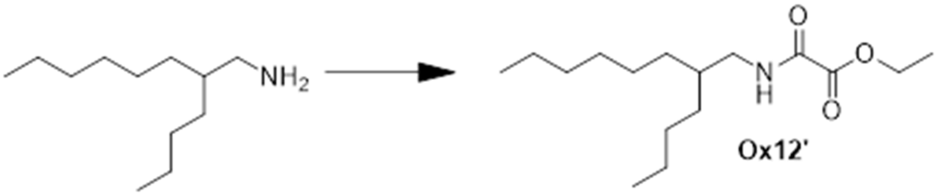

Ethyl chlorooxoacetate (5.74 g, 42.08 mmol) was added to a solution of 2-butyloctylamine (7.8 g, 42.08 mmol) in anhydrous DCM (200 mL) at 0°C. Et_3N (6.81 g, 77.38 mmol) was then added and the mixture was slowly warmed to 23°C. After 3 h, the reaction was washed with water (2×25 mL) followed by brine (25 mL). The organic layer was the dried over Na_2SO_4, filtered, and the solvent removed by rotary evaporation to afford **Ox12′** as a yellow oil (9.00 g, 74%).

^{1}H NMR ($CDCl_3$, 600 MHz) 7.06 (br s, 1H), 4.32 (q, 2H, *J* = 7.2 Hz), 3.24 (t, 2H, *J* = 7.2), 1.54 (m, 1H), 1.36 (t, 3H, *J* = 7.2 Hz), 1.25-1.29 (m, 16H), 0.85-0.89 (m, 6H).

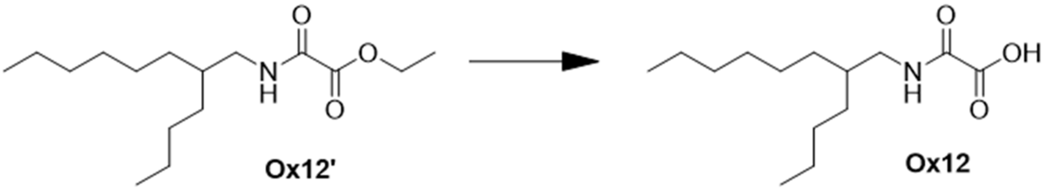

Aqueous 2.5 M NaOH (10 mL, 62.62 mmol) was added to a solution of **Ox12′** (9.00 g, 31.31 mmol) in THF (200 mL). The reaction was then stirred at 23°C for 6h before 2M HCl was added to lower the pH to ~3. The excess THF was then removed by rotary evaporation and the aqueous mixture was then extracted with DCM (3×50 mL). The DCM extracts were combined and dried over Na_2SO_4, filtered and concentrated by rotary evaporation to afford **Ox12** as a white solid (7.91 g, 98%).

^{1}H NMR ($CDCl_3$, 600 MHz) 7.26 (br s, 1H), 3.30 (t, 2H, *J* = 7.2), 1.57 (m, 1H), 1.26-1.32 (m, 16H), 0.86-0.90 (m, 6H).

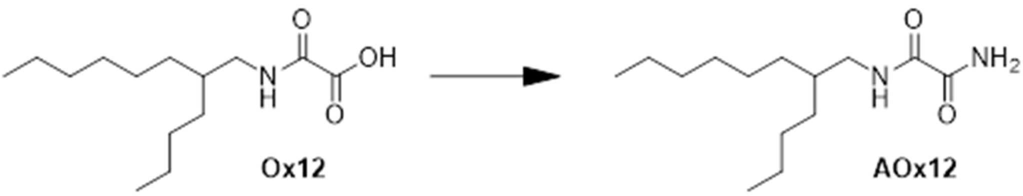

Oxalyl chloride (3.50 mL, 41.39 mmol) was added dropwise to a solution of **Ox12** (5.90 g, 23.00 mmol) in anhydrous THF (150 mL) and anhydrous DMF (0.1 mL) at 0 °C. The reaction was then slowly warmed to 23°C. After 4 h, the excess THF was removed by rotary evaporation and the crude residue was dried *in vacuo*. Fresh anhydrous THF (200 mL) was then added and the mixture was cooled to 0°C before adding aqueous NH_4OH (10~30 %, 40 mL) dropwise. The mixture was then warmed to 23°C and allowed to stir for an additional 30 min., before the excess THF was removed by rotary evaporation. The white solid residue was dispersed in deionized water, filtered and washed with deionized water before drying *in vacuo* for 18 h to afford the compound **AOx12** as a white solid (5.10 g, 86%).

^{1}H NMR ($CDCl_3$, 600 MHz) δ 7.38 (br s, 1H), 7.33 (br s, 1H), 5.65 (br s, 1H), 3.27 (t, 2H, *J* = 6 Hz), 1.56 (m, 1H), 1.26-1.31 (m, 16H), 0.86-0.89 (m, 6H). ^{13}C NMR ($CDCl_3$, 150 MHz) δ 162.7, 160.7, 42.9, 37.4, 31.7, 31.6, 31.2, 29.5, 28.6, 26.3, 22.9, 22.5, 14.4. HRMS (ESI, M^+) cald

for $C_{14}H_{28}N_2O_3$ 279.2043 $(M+Na)^+$, 279.2044 $(M+Na)^+$ found. FT-IR (KBr) ν (cm^{-1}) 3374, 3309, 3192, 2922, 2854, 1653, 1603, 1546, 1447, 1410, 1376, 1261, 1148.

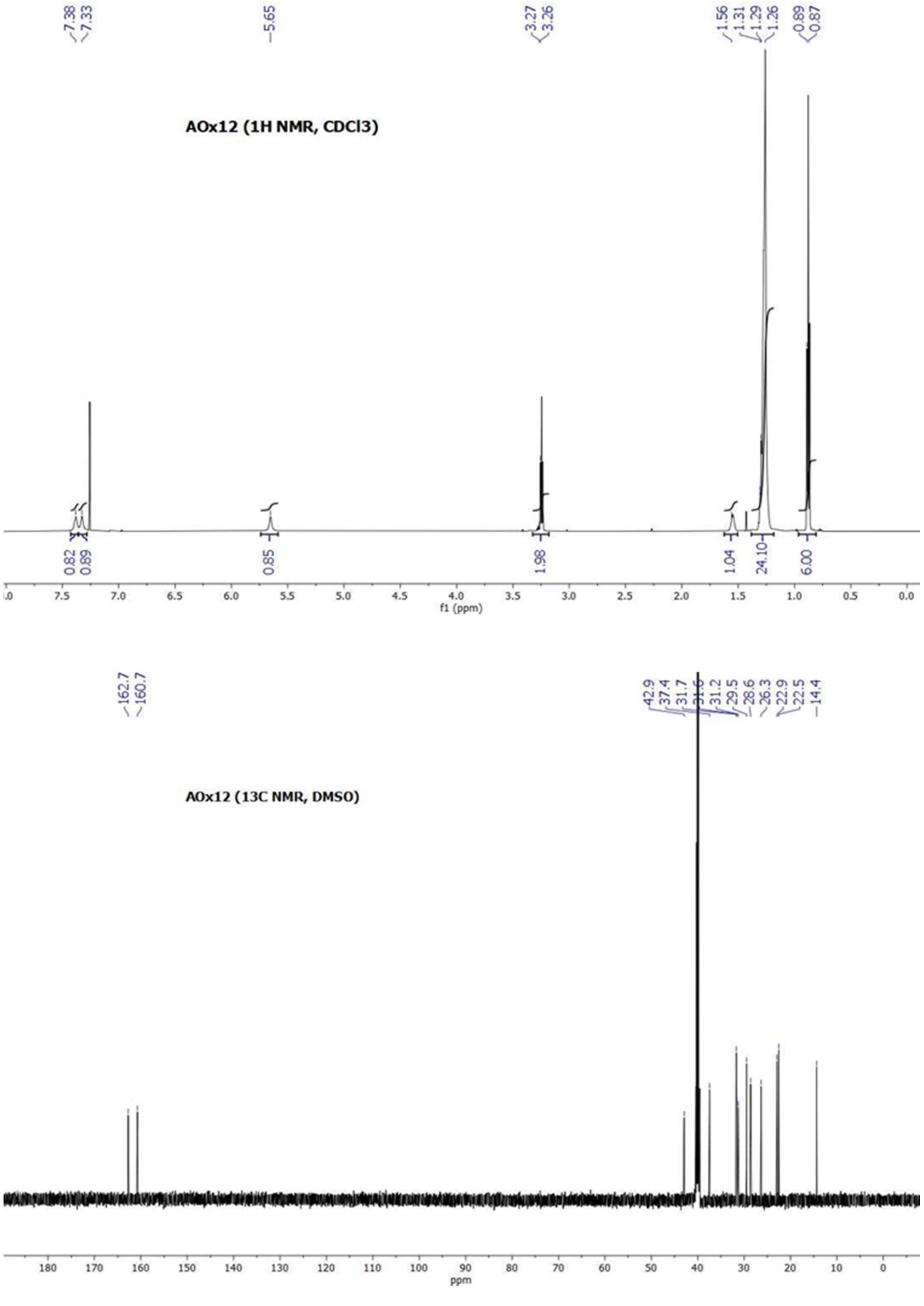

Figure S2. a. ^1H and ^{13}C NMR of **AOx12.**

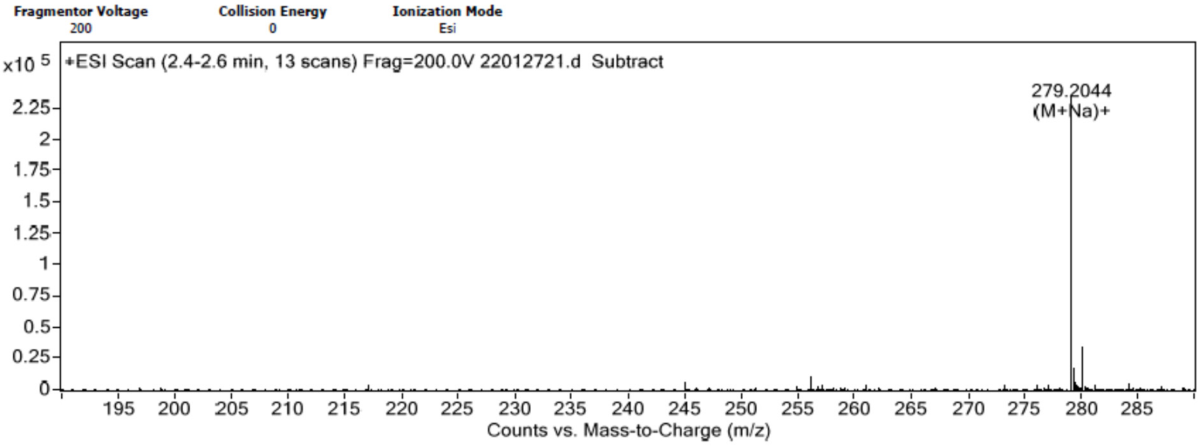

Formula Calculator Results

Formula	Ion Formula	Mass	Calc Mass	m/z	Calc. m/z	Diff (mDa)	Diff (ppm)	DBE	Ion Species	Score
C14 H28 N2 O2	C14 H28 N2 Na O2	256.2152	256.2151	279.2044	279.2043	-0.09	-0.33	2	(M+Na)+	97.97

Figure S2. b. High resolution mass spectrum of **AOx12.**

Synthesis of AOx16

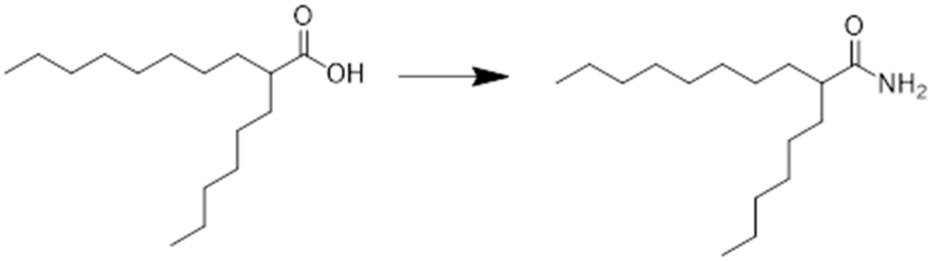

Oxalyl chloride (7.49 g, 59.0 mmol) was added dropwise to a solution of 2-hexyldecanoic acid (10 g, 38.99 mmol) and anhydrous DMF (0.1 mL) in anhydrous THF (250 mL) at 0°C. The mixture was then allowed to warm to 23°C. After 4 h, the excess THF was removed by rotary evaporation and the crude was dried *in vacuo*. Fresh THF (200 mL) was added and the mixture was cooled to 0°C before adding aqueous NH_4OH solution (10-30%, 40 mL) dropwise. The mixture was warmed to 23°C. After 30 min, the excess THF was removed by rotary evaporation and the resulting white solid was filtered and washed with water and dried *in vacuo* for 18 h to give 2-butyloctanamide as a white solid (9.95 g, 99%).

^{1}H NMR ($CDCl_3$, 600 MHz) 5.43 (br s, 2H), 2.08 (d, 1H, J = 5.4), 1.57 (m, 2H), 1.43 (m, 2H), 1.26-1.32 (m, 20H), 0.85-0.89 (m, 6H).

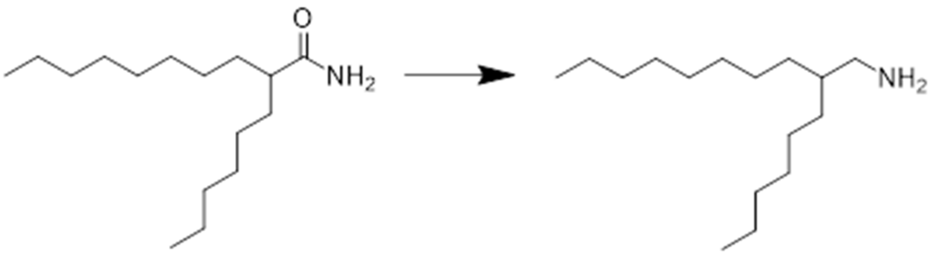

LiAlH4 (5.32 g, 140.25 mmol) was added in portions over 1 h to a solution of 2-hexyldecanamide (9.67 g, 37.88 mmol) in anhydrous THF (250 mL) at 0°C. After 1 h, the reaction was warmed to 23°C for 2 d and then refluxed for 24 h. The mixture was cooled to 0 °C and water (5.5 mL) was added dropwise over 45 min, followed by a solution of aqueous 0.63 M NaOH solution (22 mL, 13.8 mmol). After stirring at 23°C for 2 h, the mixture was filtered through celite and the excess THF was removed by rotary evaporation. The

resulting aqueous phase was then extracted with DCM (200 mL). The organic layer was separated and washed with water (2×20 mL) and brine (50 mL) before drying over Na_2SO_4, filtering, concentrating by rotary evaporation and drying *in vacuo* to afford 2-hexyldecylamine as a viscous yellow liquid (8.05 g, 88%).

^{1}H NMR ($CDCl_3$, 600 MHz) 2.58 (d, 1H, *J* = 5.4 Hz), 1.25-1.30 (m, 25H), 0.86-0.88 (m, 6H).

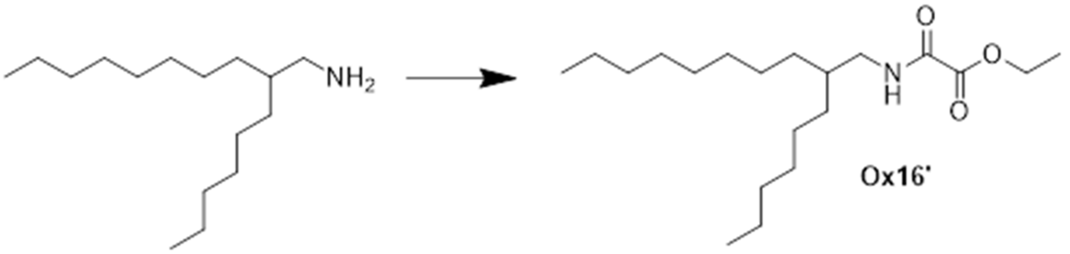

Ethyl chlorooxoacetate (4.98 g, 36.44 mmol) was added to a solution of 2-hexyldecylamine (8.00 g, 33.13 mmol) in anhydrous DCM (200 mL) at 0°C. Et_3N (6.70 g, 66.26 mmol) was then added and the mixture was slowly warmed to 23°C. After 3 h, the reaction was washed with water (2 × 25 mL) followed by brine (25 mL). The organic layer was the dried over Na_2SO_4, filtered, and the solvent removed by rotary evaporation to afford **Ox16′** as a yellow oil (7.9 g, 70%).

^{1}H NMR ($CDCl_3$, 600 MHz) 7.05 (br s, 1H), 4.33 (q, 2H, *J* = 7.2 Hz), 3.26 (t, 2H, *J* = 6 Hz), 1.54 (m, 1H), 1.38 (t, 3H, *J* = 7.2 Hz), 1.23-1.29 (m, 24H), 0.85-0.89 (m, 6H).

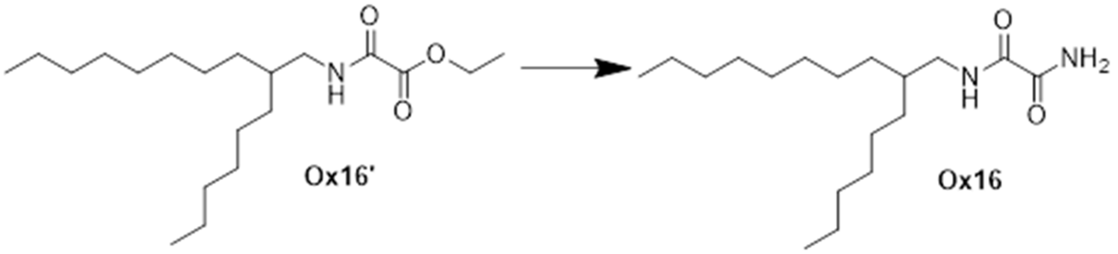

Aqueous 2.5M NaOH (18 mL, 45.67 mmol) was added to a solution of **Ox16′** (7.80 g, 22.83 mmol) in THF (200 mL). After 1 h, 2M HCl was added to lower the pH ~3. The excess THF was removed by rotary evaporation and resulting aqueous phase was extracted with DCM (3×50 mL). The combined extracts were then dried over Na_2SO_4, filtered and concentrated *in vacuo* to afford a white solid. The solid was then dissolved in fresh anhydrous THF (200 mL) and anhydrous DMF (0.1 mL) and the mixture was then cooled at 0°C before adding oxalyl chloride (3.5 mL, 41.09 mmol) dropwise. The reaction was warmed to 23°C. After stirring for 4 h, the excess THF was removed by rotary evaporation and the crude was dried *in vacuo*. The crude solid was then dissolved in fresh anhydrous THF (250 mL) and cooled at 0°C before adding aqueous NH_4OH (10~30%, 40 mL). The mixture was then allowed to stir at 23°C for 2 h. The excess THF was then removed by rotary evaporation to give a white suspension that was filtered and washed with deionized water and dried *in vacuo* for 18 h to afford the compound **AOx16** as a white solid (6.70 g, 98%).

^{1}H NMR ($CDCl_3$, 600 MHz) δ 7.38 (br s, 1H), 7.33 (br s, 1H), 5.65 (br s, 1H), 3.24 (t, 2H, *J* = 6 Hz), 1.56 (m, 1H), 1.25-1.30 (m, 24H), 0.87 (t, 6H, *J* = 6.6 Hz). ^{13}C NMR ($CDCl_3$, 150 MHz) δ 162.1, 159.3, 43.0, 37.8, 31.8, 31.7, 29.9, 29.5, 29.3, 26.6, 26.5, 22.6, 14.1. HRMS (ESI, M^+) cald for $C_{18}H_{36}N_2O_2$ 335.2669 $(M+Na)^+$, 335.2670 $(M+Na)^+$ found. FT-IR (KBr) ν (cm^{-1}) 3383, 3310, 3209, 2920, 2851, 1651, 1603, 1544, 1445, 1411, 1376, 1254, 1110.

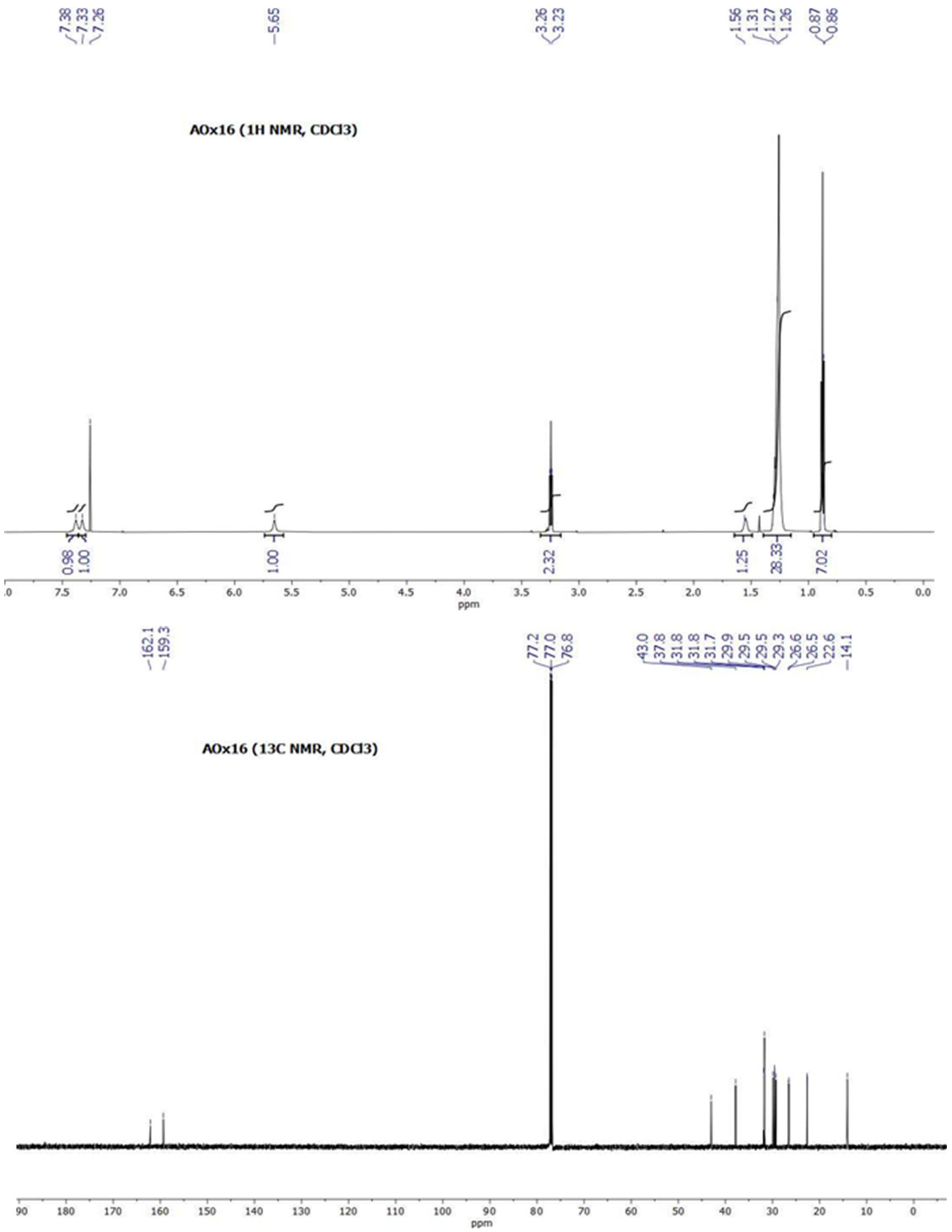

Figure S3. a. ^{1}H and ^{13}C NMR of **AOx16.**

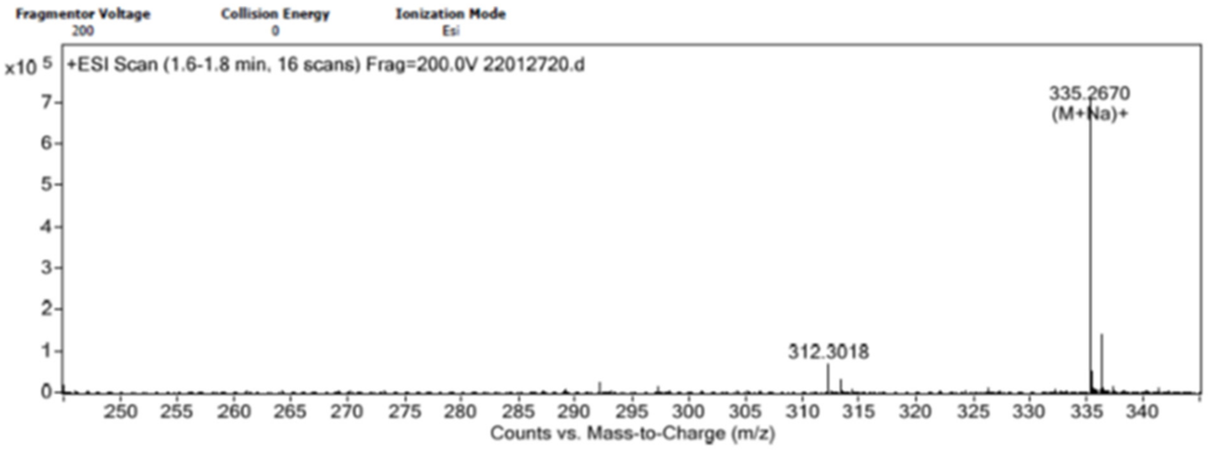

Formula Calculator Results

Formula	Ion Formula	Mass	Calc Mass	m/z	Calc. m/z	Diff (mDa)	Diff (ppm)	DBE	Ion Species	Score
C18 H36 N2 O2	C18 H36 N2 Na O2	312.2778	312.2777	335.267	335.2669	-0.08	-0.24	2	(M+Na)+	99.1

Figure S3. b. High resolution mass spectrum of **AOx16.**

Synthesis of AOx24

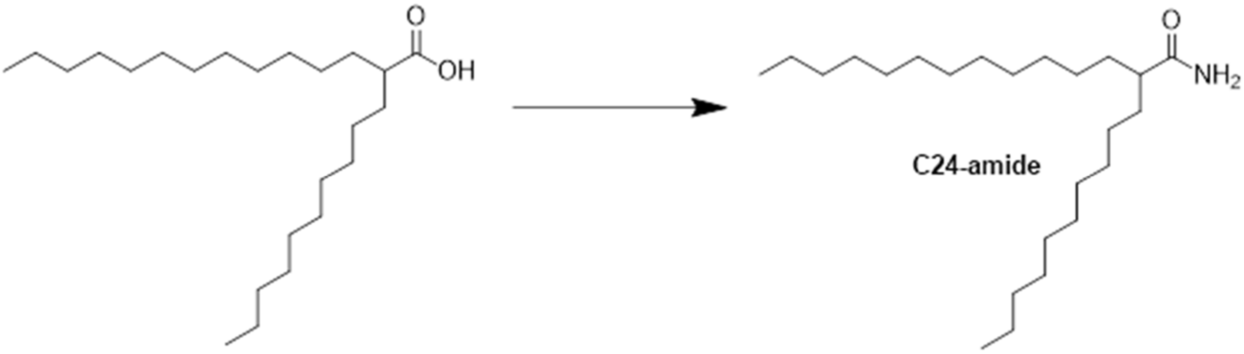

Oxalyl chloride (5.17 g, 40.77 mmol) was added dropwise to a solution of 2-decyltetradecanoic acid (10.00 g, 27.17 mmol) and anhydrous DMF (0.1 mL) in anhydrous THF (250 mL) at 0°C. The mixture was then allowed to warm to 23°C. After 18 h, the excess THF was removed by rotary evaporation and the crude was dried *in vacuo*. Fresh THF (200 mL) was added and the mixture was cooled to 0°C before adding aqueous NH_4OH solution (10-30%, 40 mL) dropwise to form a white precipitate. The mixture was warmed to 23°C. After 60 min, the resulting white solid was filtered and washed with water and THF before drying *in vacuo* for 18 hh to give **C24-amide** as a white solid (10.10 g, 99%).

^{1}H NMR (600 MHz, acetone-d_6) 6.69 (s, 1H), 6.01 (s, 1H), 2.19 (br s, 1H), 1.93 (br s, 2H), 1.54 (m, 2H), 1.27 (m, 34H), 0.87 (t, 6H).

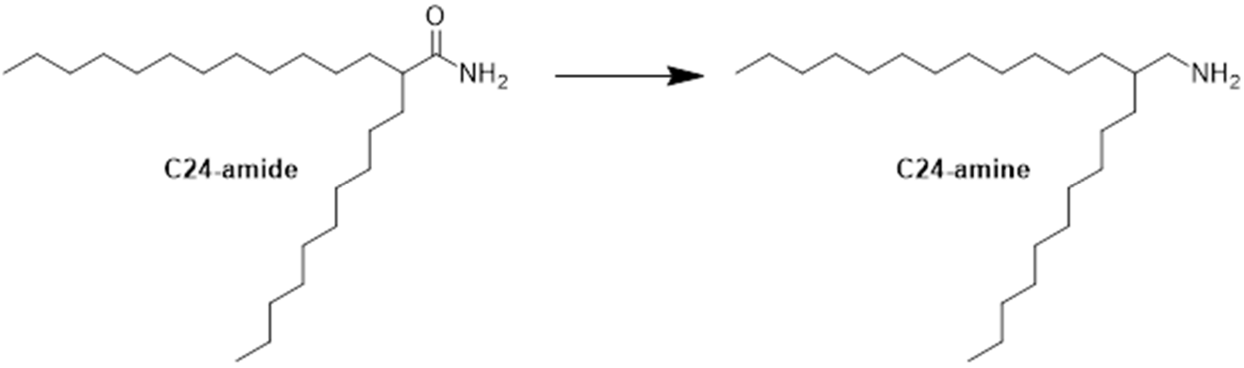

$LiAlH_4$ (3.09 g, 81.51 mmol) was added in portions over 1 h to a suspension of **C-24 amide** (10.00 g, 27.17 mmol) in anhydrous THF (200 mL) at 0°C. The mixture was allowed to stir at 23°C for 30 min. before heating to reflux. After 24 h, the mixture was cooled to 0°C and water (12.1 mL) was added dropwise followed by 2.5 M aqueous NaOH (3.1 mL). After 1 h, the mixture was slowly warmed to 23°C and stirred for an additional 3 h before filtered through Celite. The filtrate was then concentrated by rotary evaporation to afford **C-24 amine** as colorless oil (8.13 g, 85%).

^1H NMR (600 MHZ, $CDCl_3$) 2.57 (d, 1H, *J* = 5.4 Hz), 1.24-1.27 (m, 41H), 0.86 (t, 6H, *J* = 7.2 Hz).

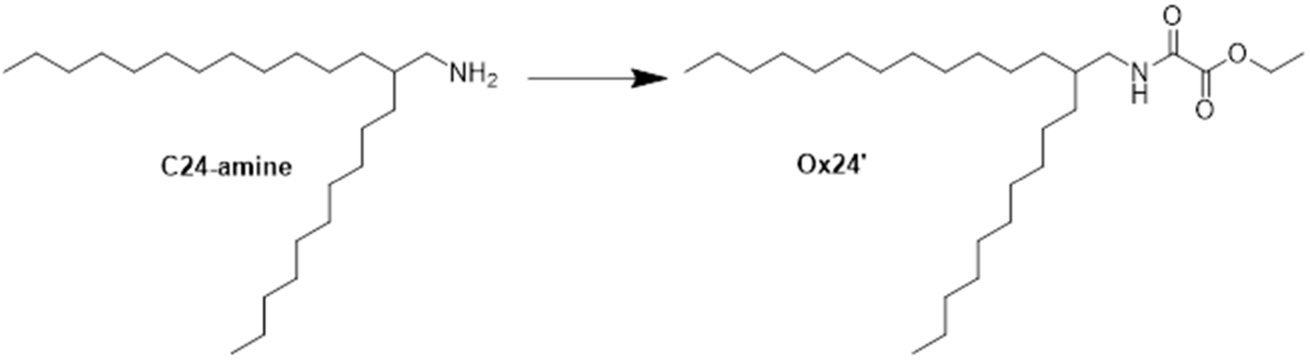

Ethyl chlorooxoacetate (1.08 g, 7.91 mmol) was added to a solution of 2-decyltetradecylamine (2.00 g, 6.58 mmol) in anhydrous DCM (50 mL) at 0°C. Et_3N (1.32 g, 13.16 mmol) was then added and the mixture was slowly warmed to 23°C. After 2 h, the excess DCM was removed by rotary evaporation. Ethyl acetate (50 mL) was added to the crude residue and the mixture was then washed water (20 mL) and brine (20 mL). The ethyl acetate layer was dried over Na_2SO_4, filtered, and concentrated by rotary evaporation to afford **Ox24′** as a yellow oil (2.95 g, 99%)

^1H NMR (600 MHz, $CDCl_3$) 7.10 (br s, 1H), 4.33 (q, 2H), 3.25 (t, 2H), 1.56 (m, 1H), 1.27 (t, 3H), 1.22-1.26 (m, 40H), 0.86 (t, 6H).

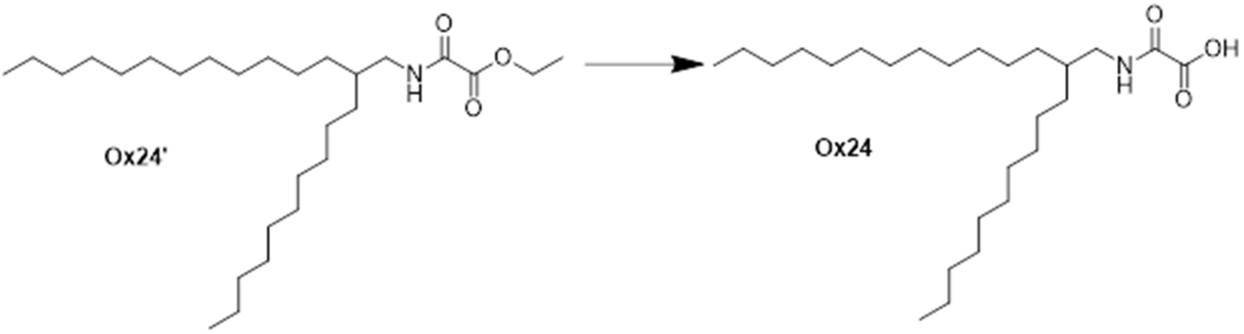

Aqueous 2.5M NaOH (0.52 g, 13 mmol) was added to a solution of **Ox24′** (2.90 g, 6.5 mmol) in THF (200 mL). After 1 h, 2M HCl was added until pH ~3. THF was removed by rotary evaporation and a white precipitate was formed, which was filtered, washed with deionized water and ethyl acetate, then dried *in vacuo* for 18 h to afford the **Ox24** as a white solid (2.72 g, 99%).

^1H NMR (600 MHz, $CDCl_3$) 3.28 (t, 2H), 1.58 (m, 1H), 1.27 (t, 3H), 1.23-1.28 (m, 40H), 0.86 (t, 6H)

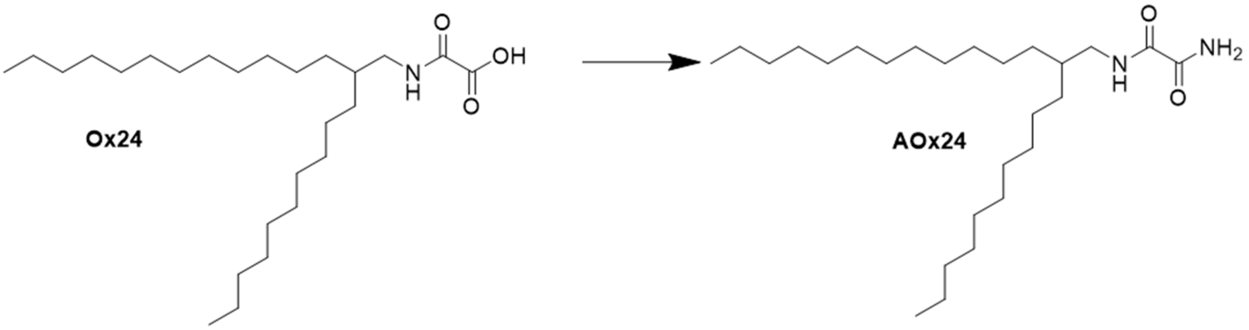

Oxalyl chloride (1.13 g, 8.94 mmol) was added dropwise to a solution of **Ox24** (1.90 g, 4.47 mmol) in anhydrous THF (100 mL) and anhydrous DMF (0.1 mL) at 0°C. The reaction was then slowly warmed to 23°C. After 4 h, the excess THF was removed by rotary evaporation and the crude residue was dried *in vacuo*. Fresh anhydrous THF (100 mL) was then added and the mixture was cooled to 0°C before adding aqueous NH_4OH (10~30%, 10 mL) dropwise. The mixture was then warmed to 23°C and allowed to stir for an additional 30 min, before the excess THF was removed by rotary evaporation. The white solid residue was dispersed in deionized water, filtered, washed with deionized water and then hexanes and diethyl ether before drying *in vacuo* for 18 h to afford the compound **AOx24** as a white solid (1.58 g, 83 %).

^{1}H NMR (600 MHz, $CDCl_3$) δ 7.36 (br s, 1H), 7.30 (br s, 1H), 5.58 (br s, 1H), 3.24 (t, 2H, *J* = 7.2 Hz) 1.55 (m, 1H), 1.26-1.31 (m, 40H), 0.86 (t, 6H, *J* = 7.2 Hz). ^{13}C NMR (150 MHz, $CDCl_3$) δ 162.0, 159.3, 43.0, 37.8, 31.9 (x2), 31.7, 29.9, 29.7, 29.6 (x5), 29.3 (x2), 26.6, 22.7, 14.1. HRMS (ESI, M^+) cald for $C_{26}H_{52}N_2O_2$ 447.3921 $(M+Na)^+$, 447.3922 $(M+Na)^+$ found. Elemental analysis: calculated (%) for $C_{26}H_{52}N_2O_2$, C: 73.5, H: 12.3, N: 6.6 found (%), C: 73.4, H: 12.4, N: 6.6. FT-IR (KBr) ν (cm^{-1}) 3384, 3309, 3213, 2917, 2849, 1651, 1601, 1544, 1410, 1376, 1255, 1109.

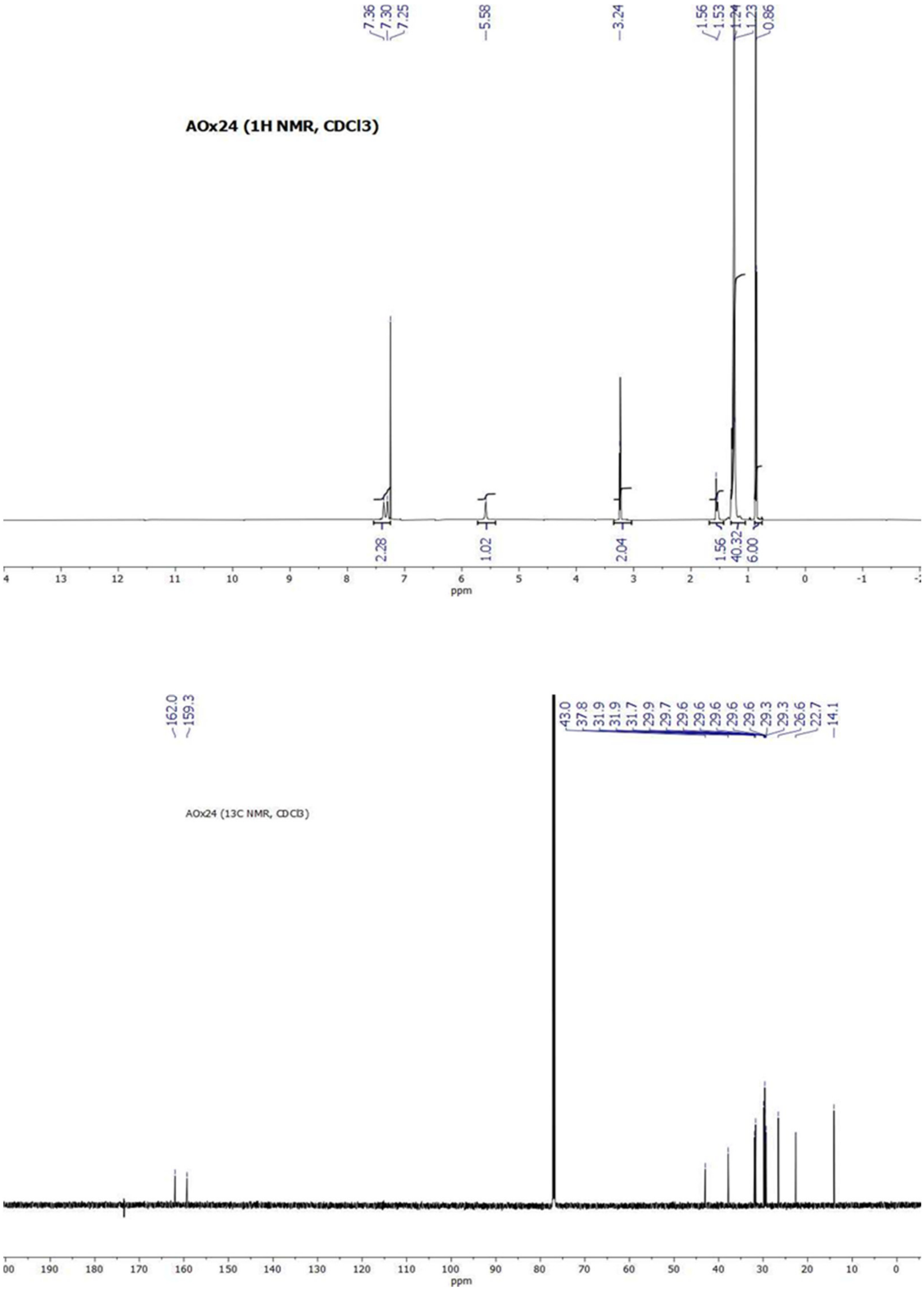

Figure S4. a. ^{1}H and ^{13}C NMR of **AOx24.**

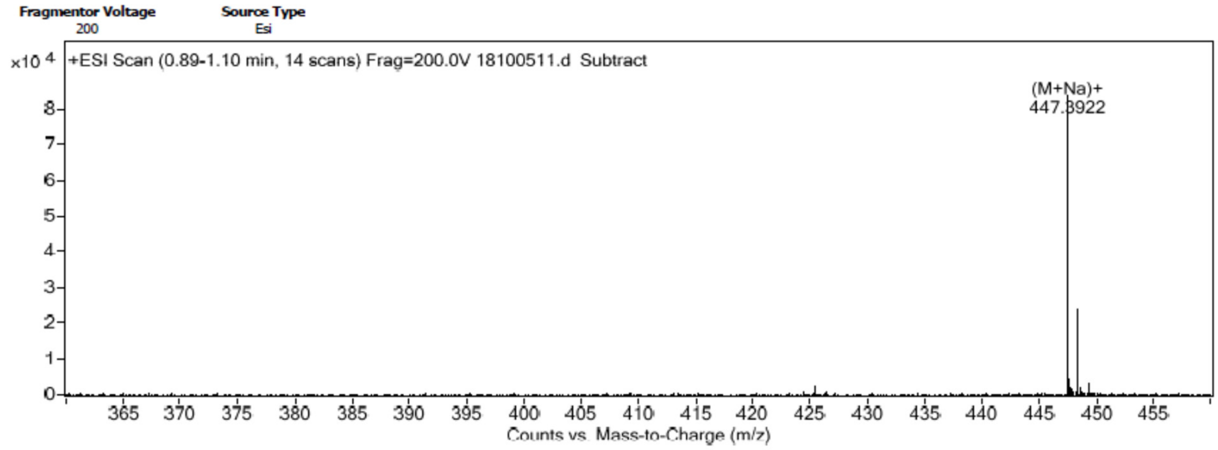

Formula Calculator Results

Formula	Ion Species	Mass	Calc. Mass	m/z	Calc. m/z	Diff (mDa)	Diff (ppm)	DBE	Ion	Score
C26 H52 N2 O2	C26 H52 N2 Na O2	424.403	424.4029	447.3922	447.3921	-0.12	-0.27	2	(M+Na)+	88.78

Figure S4. b. High resolution mass spectrum of **AOx24.**

Additional Data

Table S1. Gelator test data for alkylated oxalamides.

Copyright: © 2022 by the authors. Licensee MDPI, Basel, Switzerland. This article is an open access article distributed under the terms and conditions of the Creative Commons Attribution (CC BY) license (https://creativecommons.org/licenses/by/4.0/).

Solvent	AOx8	AOx12	AOx16	AOx24
Acetone	OG (2.2)	OG (2.2)	P	OG (2.4)
Acetonitrile	OG (2.0)	OG (2.0)	OG (0.8)	OG (0.3)
Benzyl alcohol	OG (2.3)	OG (2.3)	S	TG (0.8)
nButanol	OG (1.0)	OG (1.9)	OG (2.8)	OG (2.0)
Castor oil	TG (0.8)	CG (0.8)	OG (0.7)	CG (0.7)
Chloroform	OG (3.3)	S	S	S
Cinnamaldehyde	OG (2.6)	OG (0.6)	OG (2.6)	OG (1.2)
Corn oil	TG (<0.4)	TG (<0.4)	CG (<0.4)	CG (<0.4)
Cyclohexane	OG (2.0)	TG (<0.2)	CG (<0.1)	TG (0.2)
1,4-Dioxane	S	S	S	S
Dodecane	TG (0.8)	OG (<0.1)	TG (<0.1)	TG (<0.1)
DMF	S	OG (2.6)	OG (2.7)	OG (2.5)
DMSO	S	S	S	OG (2.5)
Ethanolamine	TG (0.8)	OG (1.9)	TG (0.5)	OG (0.1)
Ethyl acetate	OG (2.5)	OG (2.5)	P	OG (2.5)
Hexanes	OG (2.0)	OG (0.2)	TG (<0.1)	TG (0.1)
Hexadecane	TG (0.7)*	OG (0.05)	TG (<0.05)	TG (<0.05)
Isopropyl Myristate	OG (<1.0)	OG (<1.0)	TG (<1.0)	OG (<1.0)
Lactic acid	S	OG (0.8)	OG (<0.4)	TG (0.1)
MeOH	OG (1.8)	OG (3.0)	OG (3.0)	OG (0.3)
Methyl ethyl ketone	S	S	S	S

Oleic acid	S	S	S	S
Olive oil	TG (0.4)	TG (<0.4)	CG (<0.4)	TG (<0.4)
Propylene carbonate	OG (3.0)	OG (0.8)	OG (0.3)	OG (0.1)
Propylene glycol	OG (2.5)	OG (0.8)	TG (0.4)	OG (0.1)
Sesame oil	TG (<0.4)	TG (<0.4)	CG (<0.4)	TG (<0.4)
Soybean oil	TG (<0.4)	TG (<0.4)	CG (<0.4)	TG (<0.4)
THF	S	S	S	S
Toluene	OG (2.0)	OG (1.9)	CG (0.5)	CG (0.5)
Water	I	I	I	I
Xylenes	OG (2.4)	OG (2.2)	CG (0.5)	CG (0.5)

OG = opaque gel. S = solution. P = precipitate. TG = turbid gel. PG = partial gel. CG = clear gel. Minimum gel concentrations (MGCs) are shown in parenthesis in wt%/v. *unstable after several days. ^{s} = underwent syneresis over several days.

Table S2. Hansen Solubility Parameters (HSPs) and outcomes of solubility/gelation tests with alkylated oxalamides.

Solvent	δ_p	δ_h	$2\delta_d$	AOx8		AOx12		AOx16		AOx24	
				1 wt%	3.5 wt%	1 wt%	3.5 wt%	1 wt%	3.5 wt%	1 wt%	3.5 wt%
Acetone	10.4	7	31	S	G	PG	G	S	P	S	G
Acetonitrile	18	6.1	30.6	PG	G	PG	G	G	G	G	G
Benzyl alcohol	6.3	13.7	36.8	S	G	S	G	S	S	G	G
Butanol	5.7	15.8	32	G	G	S	G	S	G	S	G
Castor oil	4.6	12	31.8	G	G	G	G	G	G	G	G
Chloroform	3.1	5.7	35.6	S	G	S	S	S	S	S	S
Cinnamaldehyde	12.4	6.2	38	S	G	G	G	S	G	PG	G
Cyclohexane	0	0.2	33.6	PG	G	G	G	G	G	G	G
Dimethyl formamide	13.7	11.3	34.8	S	S	S	G	S	G	S	G
Dimethylsulfoxide	16.4	10.2	36.8	S	S	S	S	S	S	S	G
Dioxane	1.8	9	35	S	S	S	S	S	S	S	S
Dodecane	0	0	32	G	G	G	G	G	G	G	G
Ethanolamine	15.5	21	34	G	G	PG	G	G	G	G	G
Ethyl acetate	5.3	7.2	31.6	S	G	PG	G	P	P	S	G
Hexadecane	0	0	32.6	G	G	G	G	G	G	G	G
Hexane	0	0	29.8	PG	G	G	G	G	G	G	G
Lactic acid	8.3	28.4	34	S	S	G	G	G	G	G	G
MeOH	12.3	22.3	29.4	S	G	S	G	S	G	G	G
Oleic acid	2.8	6.2	32	S	S	S	S	S	S	S	S
Olive oil	1.2	5.4	33	G	G	G	G	G	G	G	G
Propylene carbonate	18	4.1	40	PG	G	G	G	G	G	G	G
Propylene glycol	10.4	21.3	33.6	S	G	G	G	G	G	G	G
Sesame oil	3.5	3.09	35.38	G	G	G	G	G	G	G	G
Soybean oil	2	2.7	33	G	G	G	G	G	G	G	G
THF	5.7	8	33.6	S	S	S	S	S	S	S	S
Toluene	1.4	2	36	PG	G	PG	G	G	G	G	G
water	16	42.3	31	I	I	I	I	I	I	I	I
Xylenes	1	3.1	35.2	PG	G	I	G	G	G	G	G

Gel (G), Soluble (S), Insoluble (I) for insoluble and precipitates, and Partial Gels (PG). δ_p, δ_h and $2\delta_d$ are in $MPa^{1/2.}$

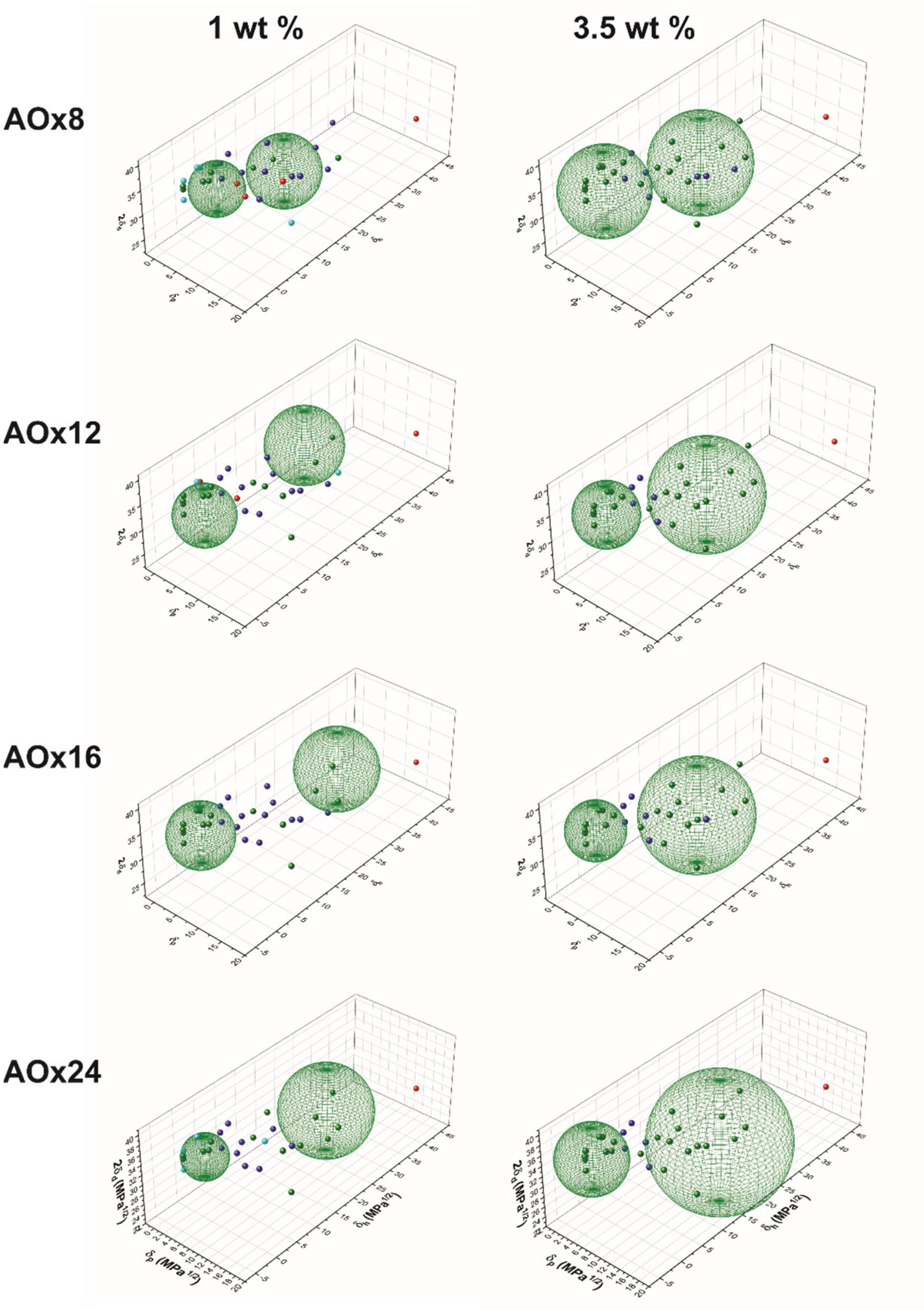

Figure S5. 3D Hansen space for gelation spheres (green) for alkyloxalamido gelators.

Table S3. Coordinates of the Centers of the Solution (S) and Gelation Spheres (G1, G2) in Hansen Space and Their Radii.

	G1				G2			
**Gelator**	δ_p (MPa$^{1/2}$)	δ_h (MPa$^{1/2}$)	$2\delta_d$ (MPa$^{1/2}$)	Radius (MPa$^{1/2}$)	δ_p (MPa$^{1/2}$)	δ_h (MPa$^{1/2}$)	$2\delta_d$ (MPa$^{1/2}$)	Radius (MPa$^{1/2}$)
**1 wt %**								
**AOx8**	4.12	1.15	32.02	4.72	7.69	16.32	30.22	6.45
**AOx12**	3.62	1.17	30.72	5.35	7.68	21.57	35.66	6.84
**AOx16**	3.56	0.47	32.90	5.70	11.74	25.18	36.60	7.29
**AOx24**	2.62	2.41	32.30	4.04	11.63	22.52	34.82	8.20

**3.5 wt %**								
**AOx8**	3.46	0.85	32.92	7.49	9.15	17.38	31.90	8.67
**AOx12**	2.01	0.82	32.46	5.46	10.24	15.51	32.04	9.57
**AOx16**	1.16	1.18	32.36	4.96	10.01	15.42	31.60	9.59
**AOx24**	1.50	0.00	32.80	6.00	16.20	13.70	35.40	11.70

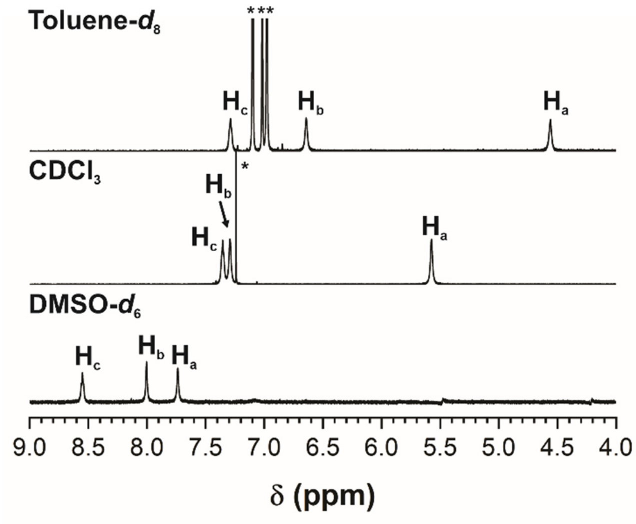

Figure S6. ^{1}H NMR spectra (600 MHz) of the amide region for **AOx24** in different solvents.

Table S4. ^{1}H NMR chemical shifts (ppm) of H_a and H_b for alkyloxalamides (20 mM) in $CDCl_3$, DMSO-d_6 and toluene-d_8 at 23°C.

**Solvent**	**DMSO-d_6**				**Toluene-d_8**				**$CDCl_3$**			
**Compound**	**H_c**	**H_b**	**H_a**	**$\Delta\delta_{ba}$**	**H_c**	**H_b**	**H_a**	**$\Delta\delta_{ba}$**	**H_c**	**H_b**	**H_a**	**$\Delta\delta_{ba}$**
**AOx8**	8.52	7.99	7.71	0.28	7.38	6.56	4.38	2.18	7.37	7.30	5.57	1.73
**AOx12**	8.53	7.99	7.71	0.28	7.23	6.55	4.33	2.22	7.31	7.29	5.56	1.73
**AOx16**	8.54	7.99	7.71	0.28	7.30	6.69	4.69	2.00	7.30	7.30	5.57	1.73
**AOx24**	8.52	7.98	7.71	0.28	7.29	6.64	4.55	1.09	7.36	7.30	5.58	1.72

*$\Delta\delta_{ba} = \delta H_b - \delta H_a$

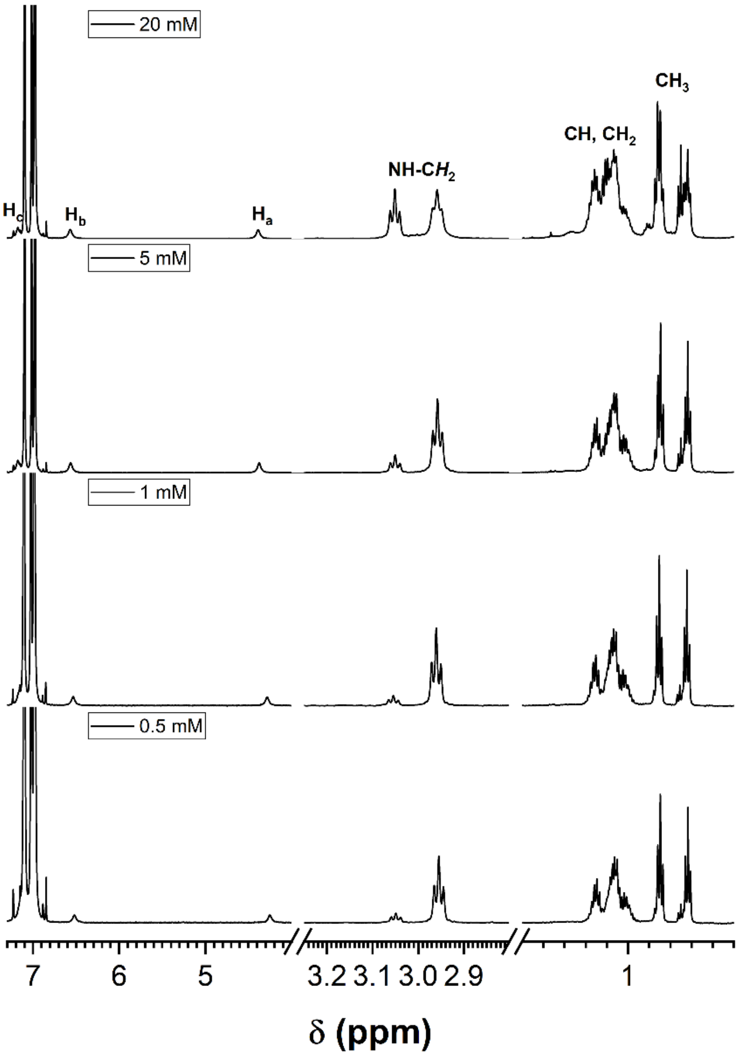

Figure S7. ^{1}H NMR (600 MHz) spectra of **AOx8** in toluene-d_8 from 0.5-50 mM.

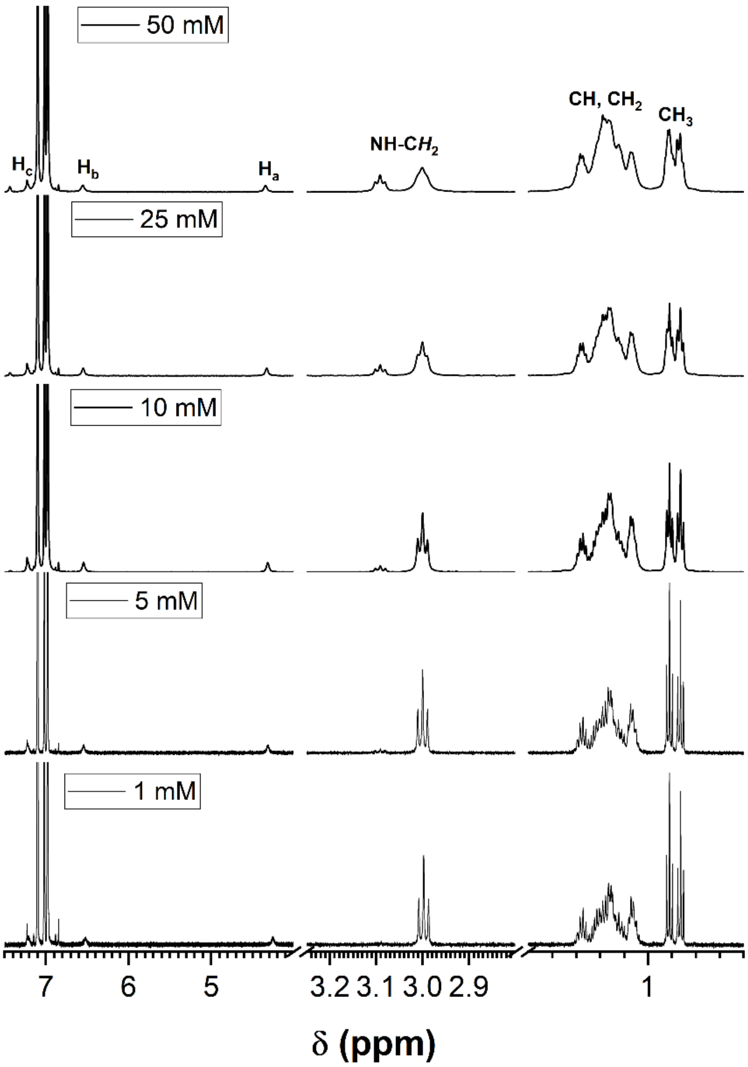

Figure S8. ^{1}H NMR (600 MHz) spectra of **AOx12** in toluene-d_8 from 1-50 mM.

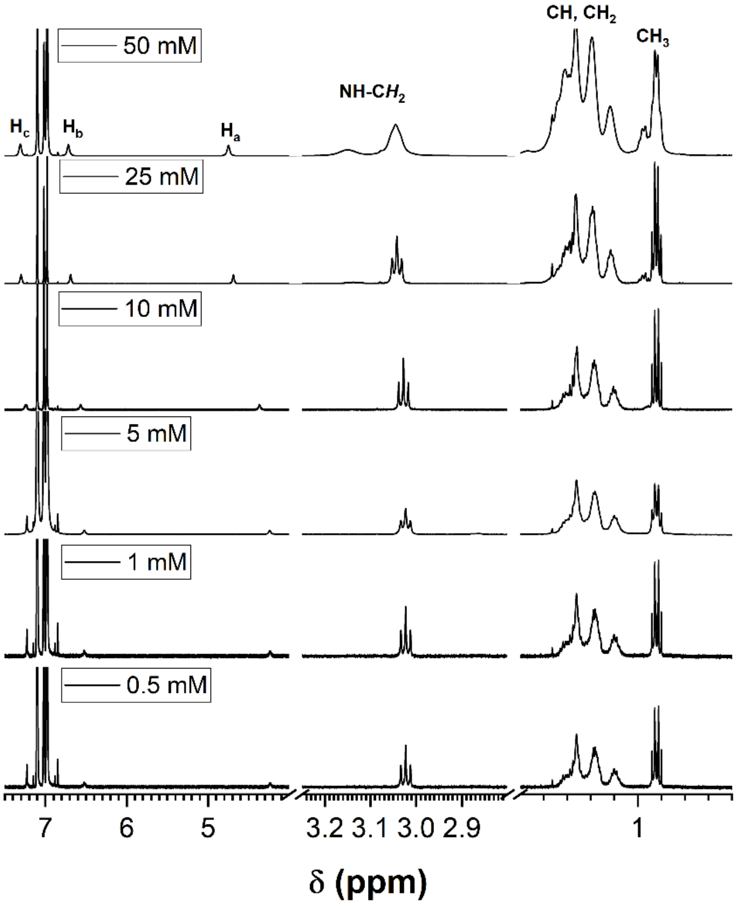

Figure S9. ^{1}H NMR (600 MHz) spectra of **AOx16** in toluene-*d*8 from 0.5-50 mM.

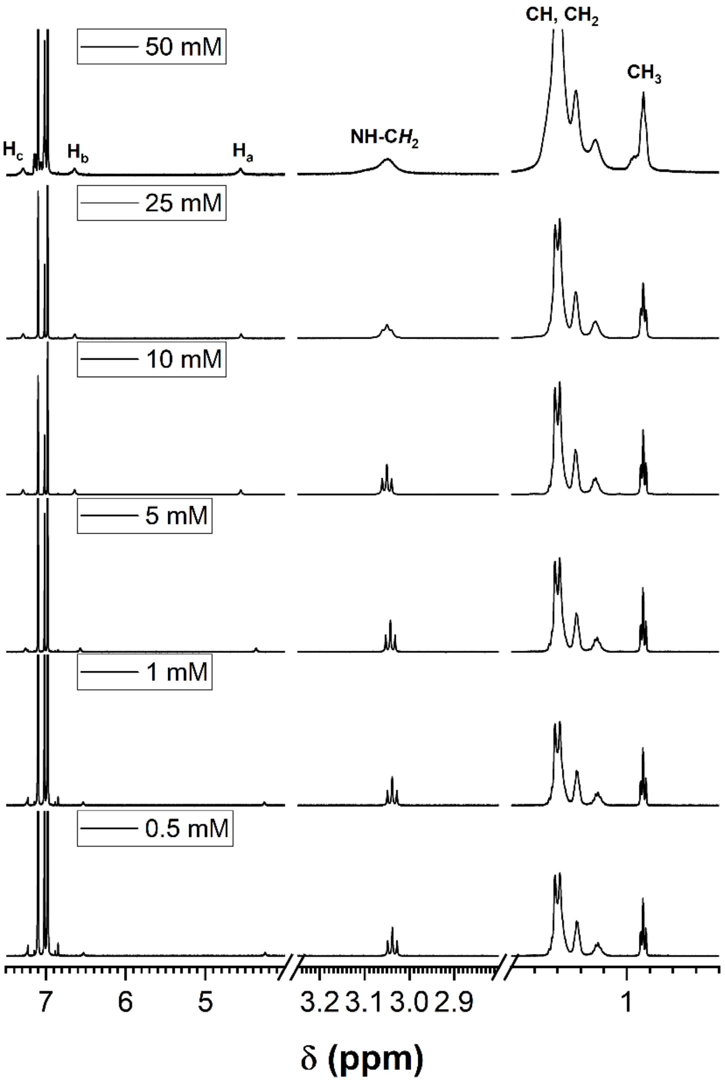

Figure S10. ^{1}H NMR (600 MHz) spectra of **AOx24** in toluene-d_8 from 0.5-50 mM. Note that the 50 mM sample was a clear gel.

Table S5. FTIR data for alkylated oxalamide gelators.

Sample	N-H str (cm^{-1})	C=O str (cm^{-1})
**AOx8**		
Powder	3373m, 3309m, 3186m	1699w, 1653s
Xerogel (Toluene)	3373m, 3309m, 3182m	1699w, 1651s
$CHCl_3$, soln	3514m, 3467w, 3396m, 3315w	1718w, 1685s, 1653w
Toluene, soln	3438w, 3377m, 3313m	3438w, 3377m, 3313m
DMSO		1675
**AOx12**		
Powder	3375m, 3311m, 3190w	1699w, 1653s
Xerogel (Toluene)	3381m, 3311m, 3203m	1716w, 1695w, 1653s
$CHCl_3$, soln	3514m, 3464w, 3394m, 3313m	1685m, 1655w
Toluene, soln	3502w, 3379m, 3309m, 3197w	1689w, 1655s
DMSO		1676
**AOx16**		
Powder	3386m, 3313m, 3209m	1693w, 1651m

Xerogel (Toluene)	3384m, 3311m, 3205m	1695w, 1651s
$CHCl_3$, soln	3514m, 3460w, 3394m, 3313w	1720w, 1685s, 1655w
Toluene, soln	3595m, 3498m, 3386m, 3317w	1720m, 1689s, 1651m, br sh
DMSO		1677
**AOx24**		
Powder	3384m, 3309m, 3213w	1653s
Xerogel (Toluene)	3384m, 3307m, 3215w	1653s
$CHCl_3$, soln	3514m, 3394m, 3309w, 3282w	1716w, 1685s, 1651w
Toluene, soln	3498m, 3386m, 3313m, 3213w	1720w, 1689s, 1655m
DMSO		1655

Soln = solution. All solutions were 0.5 wt %. s = strong. m = medium. w = weak. br sh = broad shoulder.

Table S6. Gel melting point T_{gel} of gelators in hexadecane and propylene glycol at 1 and 2 wt % from inverted vial and rheology temperature sweep experiments.

Gelator	Hexadecane (°C)			Propylene glycol (°C)		
	Rheology	Inverted Vial		Rheology	Inverted Vial	
	1 wt%	1 wt%	2 wt%	1 wt%	1 wt%	2 wt%
**AOx8**	96	90	131	-	-	-
**AOx12**	110	87	109	66	57	73
**AOx16**	114	87	94	64	68	81
**AOx24**	81	68	70	99	70	84

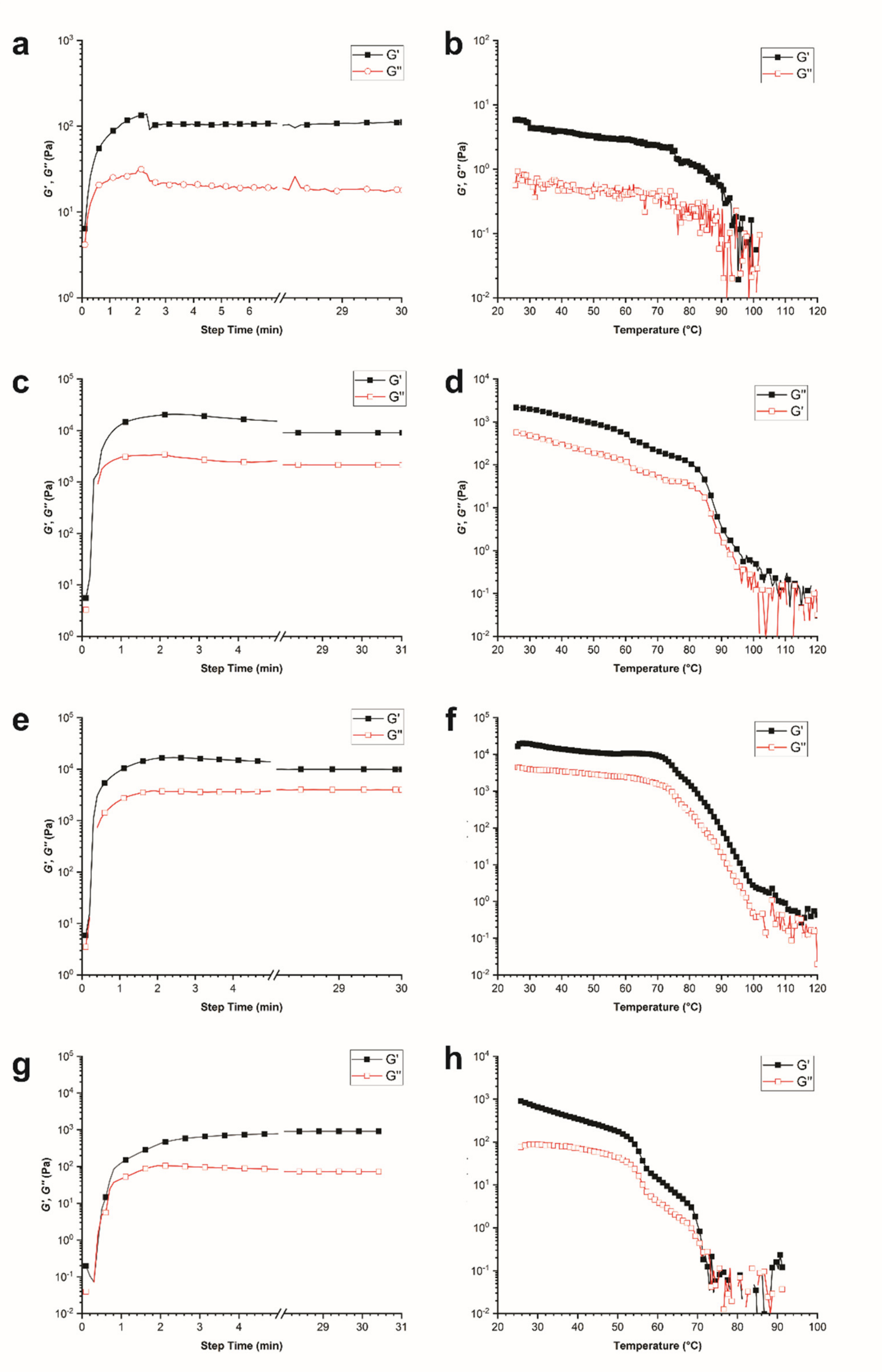

Figure S11. Rheology gel time and temperature sweep experiments for organogels of **AOx8** (a, b). **AOx12** (c, d), **AOx16** (e, f) and **AOx24** (g, h) with hexadecane.

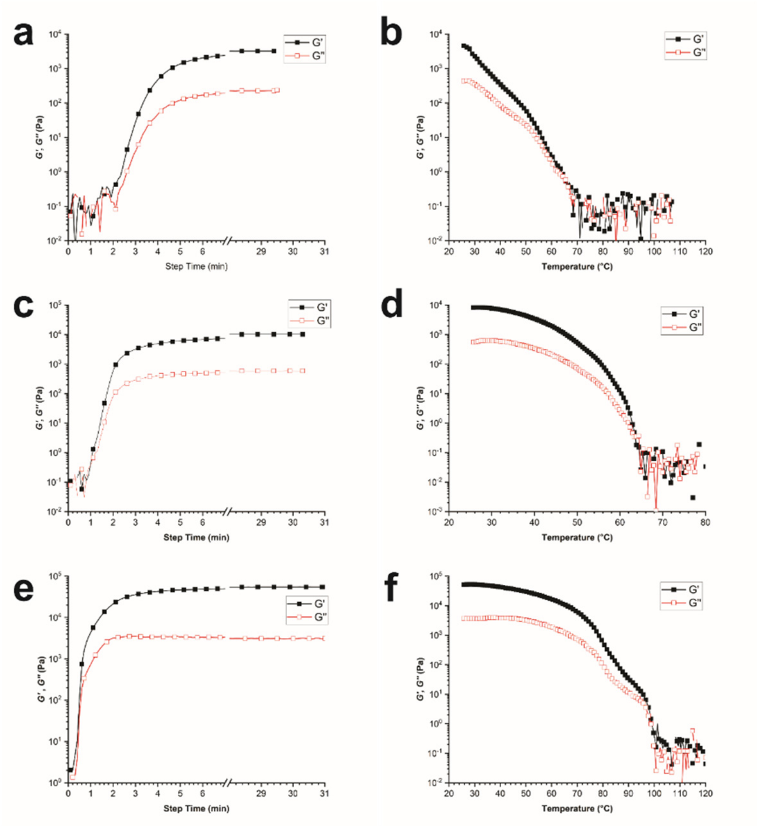

Figure S12. Rheology gel time and temperature sweep experiments organogels of **AOx12** (a, b), **AOx16** (c, d) and **AOx24** (e, f) with propylene glycol.

Table S7. Rheological properties of organogels with hexadecane (HD) and propylene glycol (PG) at 1 wt % at 25°C.

Gelator	Solvent	γ_c (%)	Crossover Strain (%) $G' = G''$	G' (Pa)	G'' (Pa)	G'/G''	$\tan\delta$ (G''/G')
**AOx8**	HD	0.20	25	105	18	5.63	0.171
**AOx12**	HD	0.48	55	9230	2517	3.7	0.273
**AOx16**	HD	0.88	45	10714	4462	2.4	0.416
**AOx24**	HD	1.33	55	945	70	13.5	0.074
**AOx8**	PG	-	-	-	-	-	-
**AOx12**	PG	0.60	10	2600	244	10.7	0.094
**AOx16**	PG	0.60	~100	10728	653	16.4	0.061
**AOx24**	PG	1.50	~100	56796	3093	18.4	0.054

γ_c = critical strain. G', G'', and G'/G'' ratios at strain = 0.15% and frequency = 10 rad/s. *Gel concentration was 1.5 wt %, since the gel at 1 wt % was not stable at larger scales. The gel underwent syneresis over several hours.

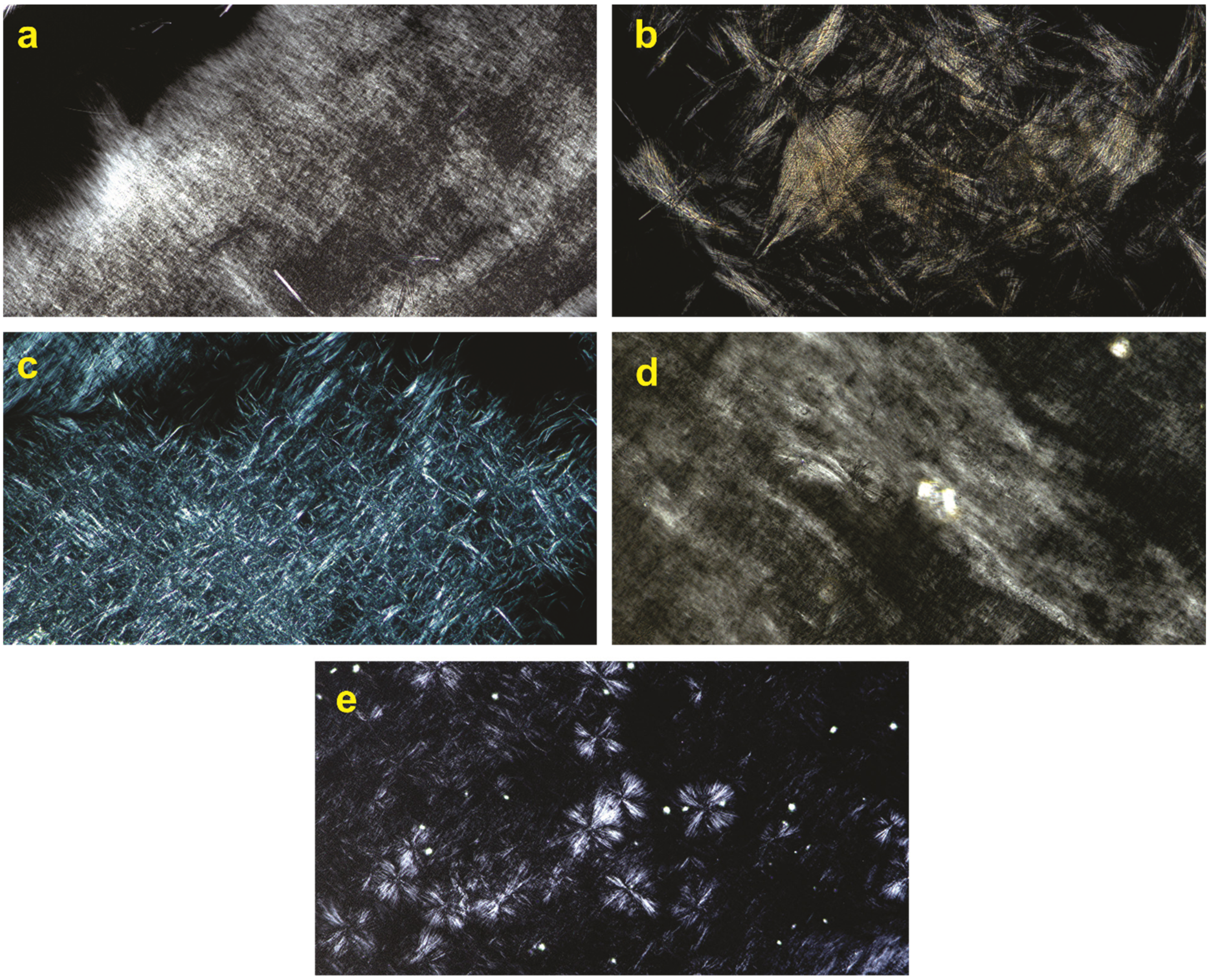

Figure S13. Polarized optical micrographs of gels from alkyloxalamides with hexadecane at 1 wt % after 1 month. (a) and (b) **AOx12**. (c) **AOx16**. (d) and (e) **AOx24**. The images are 1.42 µm wide.

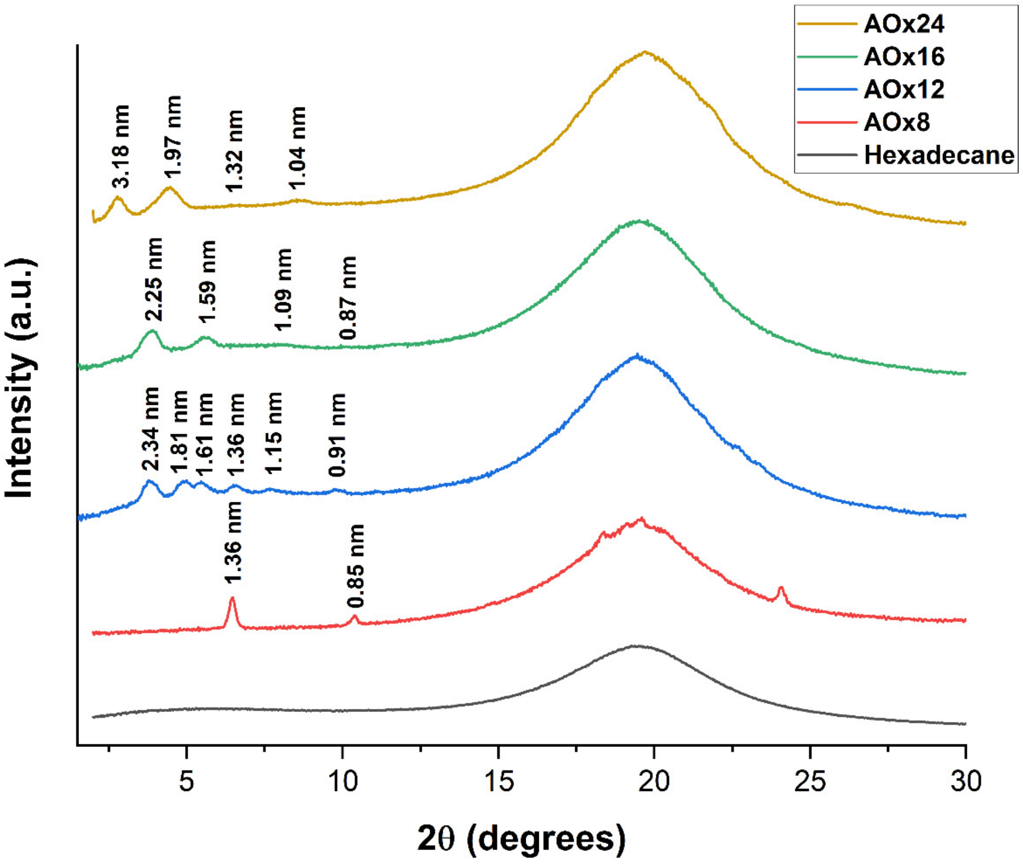

Figure S14. Powder X-Ray diffraction patterns of organogels of alkyloxalamide gelators with hexadecane. All gelator concentrations were 3 wt % except for **AOx8**, which was 1 wt %.

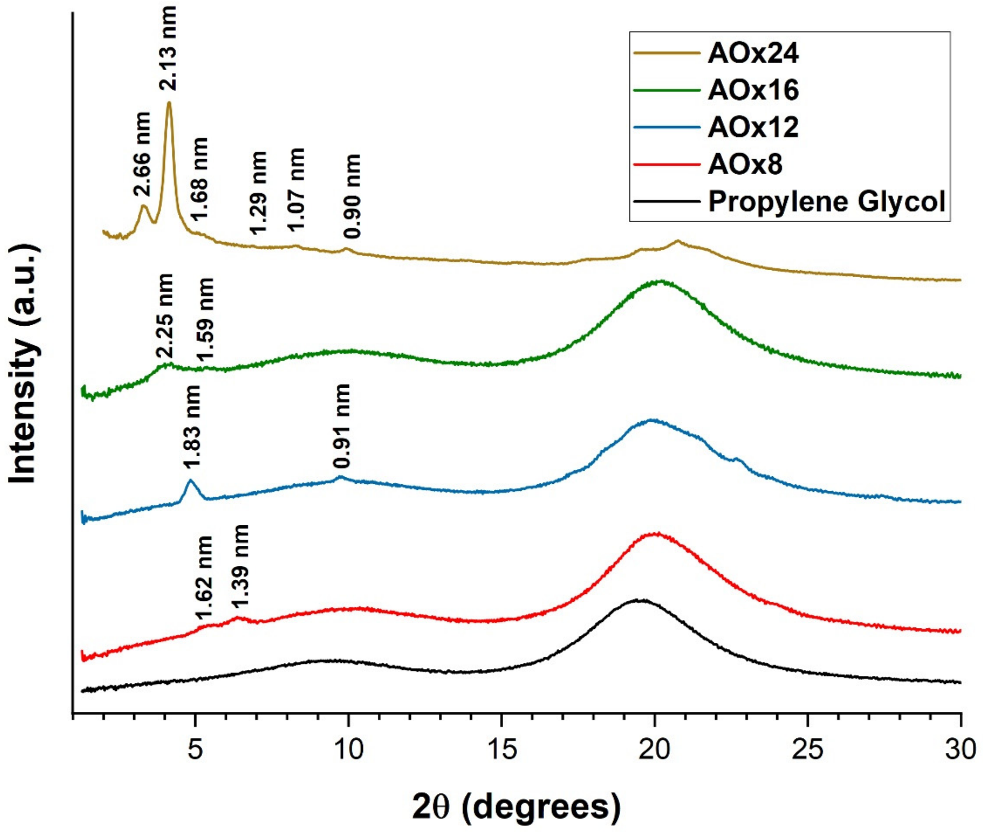

Figure S15. Powder X-Ray diffraction patterns of organogels of alkyloxalamide gelators with propylene glycol. All gelator concentrations were 5 wt %.

Disclaimer/Publisher's Note: The statements, opinions and data contained in all publications are solely those of the individual author(s) and contributor(s) and not of MDPI and/or the editor(s). MDPI and/or the editor(s) disclaim responsibility for any injury to people or property resulting from any ideas, methods, instructions or products referred to in the content.
